# Novel Diagnostic and Therapeutic Strategies in Juvenile Autoimmune Hepatitis

**DOI:** 10.3389/fped.2019.00382

**Published:** 2019-09-20

**Authors:** Marco Sciveres, Silvia Nastasio, Giuseppe Maggiore

**Affiliations:** ^1^Pediatric Hepatology and Liver Transplantation, ISMETT-University of Pittsburgh Medical Center Italy, Palermo, Italy; ^2^Division of Gastroenterology, Hepatology, and Nutrition, Harvard Medical School, Boston Children's Hospital, Boston, MA, United States; ^3^Section of Pediatrics, Department of Medical Sciences, University of Ferrara, Ferrara, Italy

**Keywords:** autoimmune liver diseases, autoimmune hepatitis, immunosuppressive therapy, B cell depletion, Tregs, monoclonal antibodies

## Abstract

Juvenile autoimmune hepatitis (JAIH) is a rare, chronic, inflammatory disease of the liver characterized by a complex interaction between genetic, immunological, and environmental factors leading to loss of immunotolerance to hepatic antigens. It affects both children and adolescents, most commonly females, and its clinical manifestations are quite variable. JAIH is progressive in nature and if left untreated may lead to cirrhosis and terminal liver failure. Although JAIH was first described almost 50 years ago, there have been few significant advances in the clinical management of these patients, both in terms of available diagnostic tools and therapeutic options. Aminotransferase activity, class G immunoglobulins and autoantibodies are the biomarkers used to diagnose AIH and monitor treatment response alongside clinical and histological findings. Despite their utility and cost-effectiveness, these biomarkers are neither an accurate expression of AIH pathogenic mechanism nor a precise measure of treatment response. Current standard of care is mainly based on the administration of steroids and azathioprine. This combination of drugs has been proven effective in inducing remission of disease in the majority of patients dramatically improving their survival; however, it not only fails to restore tolerance to hepatic autoantigens, but it also does not halt disease progression in some patients, it is often needed life-long and finally, it has deleterious side-effects. The ideal therapy should be enough selective to contrast immune-mediated live damage while preserving or potentiating the ability to develop permanent tolerance vs. pathogenic autoantigens. By reviewing the state of the art literature, this article highlights novel diagnostic and therapeutic strategies for managing pediatric AIH with a special focus on new strategies of immunotherapy. These promising tools could improve the diagnostic algorithm, more accurately predict disease prognosis, and provide targeted, individualized treatment.

## Introduction

Autoimmune hepatitis (AIH) is an inflammatory disease of the liver of unknown etiology characterized by a loss of immune tolerance against liver antigens, resulting in a progressive destruction of the hepatic parenchyma and leading, if untreated, to end-stage liver disease. It affects all ages with a peak of incidence in pediatric age, where it is referred to as Juvenile Autoimmune Hepatitis (JAIH) (1). JAIH follows a chronic but fluctuating course and is characterized by female predominance, elevated serum gamma globulins, presence of circulating autoantibodies and interface hepatitis on liver histology (2).

JAIH may present at any age from infancy to adolescence with an incidence reported of 0.4 and a prevalence of 3.0 per 100,000 children, respectively (3).

JAIH is usually symptomatic, presenting primarily as acute hepatitis, with nausea, vomiting, abdominal pain and jaundice (2, 4, 5). Such presentation can represent a real acute-onset or an acute exacerbation of long-standing liver disease but fulminant or sub-fulminant form of JAIH may also be observed (6). Clinical signs of chronic liver disease or non-specific symptoms, such as fatigue or even fortuitous findings of increased serum transaminases activity in an otherwise apparently healthy child may also be types of presentation. Symptoms and signs of a wide variety of extra-hepatic autoimmune diseases could also lead to the diagnosis of a JAIH; in particular, celiac disease has been diagnosed with higher prevalence than expected in patients with JAIH (7). JAIH can also be part of a multiorgan or systemic autoimmune disease, such as Systemic Lupus Erythematosus (SLE), Autoimmune Polyendocrinopathy Syndrome (APECED), immunodysregulation polyendocrinopathy enteropathy X-linked syndrome (IPEX), lymphoprolipherative syndromes, and STAT 1 gain of function or common variable immune deficiencies (8).

### Diagnosis of Autoimmune Hepatitis

Diagnosis is often challenging and is based on a combination of clinical, biochemical, immunological and histological criteria along with the exclusion of all known causes that may share features mimicking JAIH (9). Specific circulating autoantibodies are essential biomarkers of the disease (10): AIH type 1 is characterized by anti-Smooth Muscle Antibodies (SMA) and/or anti-Nuclear Antibodies (ANA), AIH type 2 is defined by the detection of anti-Liver-Kidney Microsomal antibody type 1 (LKM1) and/or of anti-Liver-Cytosol antibody type 1 (LC1) (11). ANA are directed against multiple nuclear antigens; ASMA recognizes mainly actin filaments (F-actin); anti LKM1 are directed against the Cytochrome P450 2D6 (CYP2D6) in the endoplasmic reticulum of hepatocytes (12); anti LC1 recognizes the Formimino-Transferase Cyclodeaminase (FTCD) in the hepatocytes cytosol (13). LKM1 and LC1 are the most specific autoantibodies and are probably implicated in the pathogenesis of AIH type 2 (14, 15).

About 20% of children with clinical, biochemical, and histological feature of autoimmune hepatitis lack both typical and non-typical autoantibodies, such as anti-soluble liver antigen (anti-SLA) or atypical perinuclear anti-neutrophil cytoplasmic antibody (pANCA). These patients represent a heterogeneous group of inflammatory liver disorders, defined as “seronegative autoimmune hepatitis” (16). These children promptly respond to immunosuppressive therapy and their liver disease may relapse when immunosuppressive therapy is discontinued.

A diagnostic scoring system for AIH based on clinical, laboratory and histologic criteria was first proposed by the International Autoimmune Hepatitis Group in 1993 and then revised in 1999 (17). This scoring system can also be useful in children, replacing alkaline phosphatase with gamma glutamyl-transferase (GGT) activity (18). A simplified diagnostic score with high specificity was more recently proposed, although it has low sensitivity in children (19, 20).

**Figure 1:**
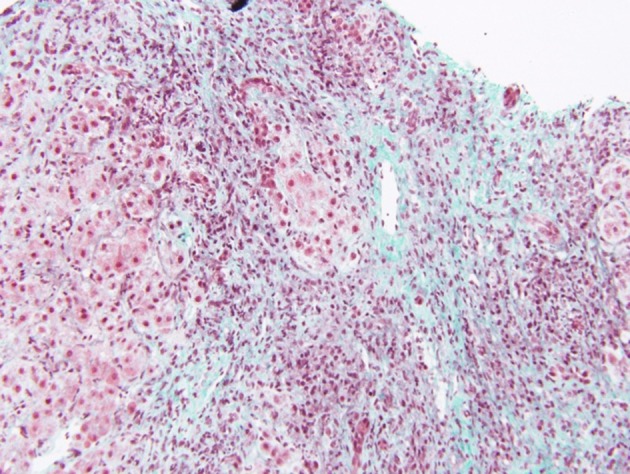
Centrolobular inflammation and fibrosis in a patient with acute presentation of AIH. Trichrome staining showing fibrosis around the central vein and lymphocytic inflammation. Bridging necrosis is also present, between the central vein and a portal tract. Disorganization of the lobular structure is evident.

Liver biopsy is often needed to establish diagnosis in non-typical cases. Histology allows grading of necro-inflammation, and staging of fibrosis. Most biopsies from children with JAIH show moderate to severe interface hepatitis and lobular inflammation (2, 5, 6) (Figure 1). Interface hepatitis consists of intense portal and periportal mononuclear cell infiltrate, consisting of CD4 and CD8 T cells, macrophages and, occasionally, eosinophils (Figure 1). Emperipolesis, based on entry of CD8 T-lymphocytes into hepatocytes is considered a highly specific feature of autoimmune hepatitis and its presence is associated with more severe necroinflammatory features and more advanced fibrosis (21).

More than half of the biopsies specimens show a variable degree of bridging fibrosis. The presence of cirrhosis at the onset ranges in published series between 40 and more than 80% of patients (2, 4–6). Although fibrosis can regress in patients with complete response to treatment, it is not clear from what stage of liver parenchyma destruction an improvement can be expected.

In presence of biliary lesions a biliary imaging is recommended to exclude main bile ducts injury, thus the possibility of autoimmune sclerosing cholangitis (ASC) (9).

### First Line Treatment

Treatment of JAIH relies on immunosuppressive therapy, administered early and for several years, to avoid relapse. The main goal of the treatment is to obtain complete remission, defined as strict normalization of serum activities of ALT/AST, and of immunoglobulin G values, as well as of the liver function (albumin, clotting factors). Prednisolone or prednisone and azathioprine constitute the so-called conventional first-line treatment of JAIH. AIH steroid-responsivity has been known for almost 5 decades now, since the first randomized, controlled treatment trials in adults documented that prednisone administered alone or in combination with azathioprine, dramatically improves symptoms, laboratory tests, histologic findings, and immediate survival (22).

Large series of children treated with conventional therapy have been reported (23–25): prednisone is usually administered orally at 2 mg/kg/day (up to a maximum of 60 mg/day), azathioprine is administered at the initial dose of 1 mg/kg/day, which can be further increased up to 2.5 mg/kg/day until sustained biochemical remission is achieved. High serum aminotransferases values and impairment of prothrombin time (PT) are considered as an indirect indicator of liver cell necrosis, i.e., severity of disease, and a guide for modulating immunosuppression. Lower doses of prednisone can be administered at the beginning of treatment to children with mild or moderate increase of liver enzymes, normal PT and a low grade of inflammation on the liver biopsy. The best timing of introduction of azathioprine is still under debate. Steroids can be started as the initial treatment with azathioprine added later. Alternatively, combination therapy can be initiated at the onset of treatment with reports of increased efficacy (26). Conventional treatment is associated with a measurable clinical and laboratory improvement within 8 weeks in over 80% of patients; however, several months may be needed to completely normalize biochemical parameters. Steroids may lead to permanent recovery of liver function, with avoidance or delay of liver transplant also in cirrhotic patients showing signs of liver failure but immunosuppression carries the risk of severe infections that are the most frequent cause of morbidity and mortality in this subgroup of patients (27). In most patients, high doses of prednisone are necessary for a relatively long period, leading to the development of harmful adverse effects. Most frequent are growth impairment and weight gain. The latter is associated with changes in the body image that often compromise the adherence to the therapeutic plan, mainly in pubertal girls and young adults (23, 28). Once remission is achieved, the aim is to maintain it in the long term with the lowest possible dose of drugs. Prednisone is progressively stopped and the patient is maintained on azathioprine monotherapy before complete discontinuation of treatment (1). The optimal duration of the immunosuppressive treatment is unknown. Current recommendation is to treat for at least 3 years, attempting withdrawal of treatment only in case of persistent and complete biochemical remission with absent or very low titer autoantibodies (9).

Relapses may occur at any time during treatment and after treatment has been discontinued, even in the absence of triggering factors.

### Current Alternative Treatments

Although conventional treatment with steroids ± azathioprine allows to achieve remission in most patients, in cases of initial treatment failure or multiple relapses during tapering or discontinuation attempts, alternative therapies are often proposed.

Cyclosporine (CSA) is a powerful immunosuppressant that has been successfully used in patients with JAIH as a short-term initial treatment alternative to steroid-azathioprine or as a salvage treatment (29, 30). The main side effects of CSA include nephrotoxicity, arterial hypertension and gastrointestinal and neurological toxicity. Minor but frequent side effects are Hypertrichosis and gum hypertrophy; although transient they occasionally can influence adherence to treatment. Since 1985, the use of CSA has been documented in 133 adults with autoimmune hepatitis. CSA was used as a salvage therapy and showed an overall positive response of any degree in about 93% of patients and a negative response, defined as no response, non-compliance, or drug intolerance in 7% (31, 32).

Regarding pediatric patients, there are only five major publications in which CSA is mostly administered for short periods, mainly to induce remission, as a bridge to a conventional treatment (29, 30, 33–35).

Furthermore, effectiveness of CSA was similar to steroids as a salvage therapy in children with JAIH and liver failure (27). Prolonged CSA treatment for autoimmune pediatric liver disorders was first reported in 2004 with an excellent safety profile (34), confirmed also in a recent follow up study (35).

A recent meta-analysis by Zizzo et al. confirmed that CSA has the highest short-term response rate (86%) in conventional treatment-refractory children with autoimmune hepatitis (36).

Besides CSA, a wide range of immunosuppressive drugs has been used in small series of children (37).

Mycophenolate Mofetil (MFM) is the second most effective drug for conventional treatment-refractory children with JAIH after cyclosporine (36% remission rate at 6 months) (36). The main concern in the use of MMF is the lack of knowledge regarding its therapeutic range, and toxic threshold; moreover, MFM is more expensive than azathioprine and is absolutely contraindicated during pregnancy.

Recently, Budesonide, a corticosteroid with potential fewer side effects than other glucocorticoids, emerged as an alternative first-line treatment in association with azathioprine. When comparing the effects of budesonide vs. prednisone, both in combination with azathioprine, budesonide did cause fewer side effects than prednisone; however, after 12 months, only 46% of the patients treated with budesonide achieved complete remission (38). The low proportion of remission observed in this study does not support its use as first-line treatment of AIH (1, 39).

mTOR inhibitors as sirolimus or everolimus have been used as salvage therapy with good results in few published adult patients (40, 41).

Liver transplantation represents a therapeutic option for a small proportion of patients under two main circumstances: patients presenting with acute liver failure that does not respond to salvage therapy with rescue immunosuppression, and patients with cirrhosis with end stage liver disease.

## Unmet Clinical Needs

### Diagnostic Issues

To this day, the diagnosis of AIH in children remains challenging and burdened by several unmet clinical needs. First of all, a highly specific single diagnostic test for the diagnosis of AIH is lacking. Furthermore, diagnosis may be complicated or potentially delayed by the fact that AIH is characterized by a very heterogeneous clinical presentation and its diagnosis requires the exclusion of all other causes of liver disease (1, 9). Class G immunoglobulins and autoantibodies are the biomarkers used to aid in the diagnosis of AIH alongside the more invasive liver biopsy. However, these biomarkers are neither an accurate expression of AIH pathogenic mechanism, nor do they univocally correlate with the degree of inflammation and fibrosis present on liver biopsy. Histology presents its own challenges, interface hepatitis is the histological hallmark of AIH but it is not exclusive to this condition and the typical histology comprising interface hepatitis, portal lymphoplasmacytic infiltrate, rosette formation, and emperipolesis, is observed in only 56% of children with autoimmune liver disease (9, 42). Unfortunately, both the scoring system created by the International Autoimmune Hepatitis Group and the simplified version proposed in 2008 have limited utility in pediatric patients (18). Moreover, AIH lacks markers and clinical tools able to provide the adequate patient risk stratification at diagnosis, which is necessary to enable providers to identify a tailored treatment. Lastly, there are no available biomarkers that might effectively guide the treatment.

In summary, main open questions are: how fast to decrease initial attack therapy (usually steroids); how long to treat before attempting to stop; when to attempt to stop treatment; risk stratification for relapse after stopping treatment. Immunoglobulins G level, presence or titer of autoantibodies and even liver histology are not predictive of success, but clinicians need to consider these elements in the absence of stronger predictors.

### Treatment Issues

Overall efficacy of available therapies along with the relative rarity of this disease has, for decades, hindered the implementation of clinical trials involving a reasonable number of patients. Although the conventional treatment with prednisone and azathioprine is associated with a high remission rate (1, 2, 23), 3 to 16% of children will undergo liver transplantation despite steroids treatment and 69 to 94% of pediatric patients will relapse after drug withdrawal (1, 2, 6). Most JAIH patients remain on treatment until adult age.

As for adult patients initial treatment failure occurs in 7–10% of patients and another 15% achieve only partial response (43). Relapse occurs after treatment withdrawal in 50–87 and 60–81% of patients become treatment-dependent (44). AIH progresses to cirrhosis in as many as 40% and 1–6% of patients with cirrhosis develop hepatocellular carcinoma (45).

The combination of these data with the evidence of steroid related side effects has constituted, and currently remains a compelling reason to refine management strategies and pursue new therapeutic options.

The main pitfall of available drugs is their complete non-selectivity. Most available immunosuppressant agents interfere indiscriminately both with the effector side of the immune system and with the regulatory compartment, thus preventing achievement of a durable desensitization against the pathogenic autoantigens, which remain unknown in most cases. However, modern techniques of molecular biology allowed for the development of powerful molecules that target single aspects of immune activation. Some of them might represent a Copernican revolution, in the field of AIH treatment, when compared with old generation immunosuppressant drugs.

## New Strategies for Diagnosis

With the advancements in molecular biology and sequencing techniques, the potential role of new biomarkers for screening and diagnosis of autoimmune hepatitis has been explored. Recent data has shown the expression of liver specific microRNAs (miRNAs) in different types of liver disorders suggesting their clinical utility as diagnostic tools for a variety of liver conditions (46). MicroRNAs (miRNAs) represent a class of endogenous, short non-coding RNAs that regulate the expression of target genes by binding to complementary regions of transcripts to repress their translation or cause mRNA degradation (47). After the presence of extracellular miRNA was discovered in body fluids, a specific miRNAs signature in different liver diseases was also described (48).

In 2015, Migita et al. investigated the circulating miRNA profiles of 46 patients with AIH and compared them to those of 40 patients with chronic hepatitis C, and 13 healthy controls. Patients were screened for 2555 miRNA using microarray and quantitative real-time PCR analysis (49). Circulating miR-21 and miR-122 were found to be significantly elevated in patients with AIH, leading to a unique miRNA expression profile. Moreover, miR-21 and miR-122 serum levels correlated with alanine aminotransferase levels and were inversely associated with increased stages of hepatic fibrosis. In contrast levels of circulating miR-21 showed a significant correlation with the histological grade of inflammation in AIH. A positive correlation between miR-21 expressed in liver tissue and ALT values was also showed in 19 patients with autoimmune liver diseases including autoimmune hepatitis, primary biliary cirrhosis, primary sclerosing cholangitis, and overlap syndrome (50).

More recently a study examined the serum miRNA profiles of 28 patients with a variety of liver disorders, including autoimmune hepatitis, as well as 4 control subjects, aiming to see whether circulating miRNAs could be used to differentiate between types of liver injury. The authors identified 37 miRNAs whose levels were different between all types of liver diseases. Interestingly, individual miRNA could not distinguish the specific type of liver disease and the authors suggest this might be due to the similarity in liver pathology beyond type of liver injury. However, the profiles of 37 miRNAs could clearly differentiate patients with liver injury from control subjects, and furthermore, could distinguish different types of liver disease (48). Nonetheless the study group is very small and includes only 4 controls and no definitive conclusions can be drawn.

The programmed death-1 receptor (PD-1, CD279) with its ligands PD-L1 (B7-H1) and PD-L2 (B7-DC) constitutes an inhibitory pathway that suppresses T cell activation and decreases lymphocyte proliferation (46). PD-1 is also found to be increased in exhausted T cells (51). PD-1, PD-L1, and PD-L2 have been detected in the liver tissue of patients with AIH and it has been hypothesized that impaired regulation of the PD-1 axis may play a role in the development of autoimmune liver diseases (52).

In 2014, a study investigated the presence of anti-PD-1 antibodies, measured by indirect enzyme-linked immunosorbent assay, in sera of 52 type 1 AIH patients, 24 patients with drug-induced liver injury (DILI), 30 patients with acute viral hepatitis (AVH), 11 patients with primary sclerosing cholangitis (PSC), and 62 healthy volunteers (53). The authors found that the prevalence of serum anti-PD-1 antibodies was greater in type 1 AIH (63%) compared to DILI (8%), AVH (13%), PSC (18%) as well as healthy controls (3%) suggesting its potential usefulness for the discrimination of type 1 AIH from other causes of liver injury. Furthermore, titers of serum anti-PD-1 antibodies were found to correlate with serum levels of bilirubin and alanine aminotransferase (ALT) but not with serum immunoglobulin G levels in type 1 AIH patients. These results were confirmed by a multicenter validation study on 71 patients with AIH and 37 patients with DILI (54). This study confirmed that serum levels of anti-PD-1 antibodies were higher in type 1 AIH patients than in DILI ones and the receiver operating curve analysis showed that measurement of anti-PD-1 antibodies was useful for the discrimination of type 1 AIH from DILI.

Although preliminary results on miRNA and PD-1 are promising, future studies are warranted to validate these findings in larger prospective studies and to demonstrate their added value and/or superiority to conventional laboratory test and histological examination.

## Perspectives for New a New Generation of Biomarkers

### Programmed Death-1 Axis

The components of PD-1 axis are potentially not only a valid auxiliary diagnostic marker for type 1 AIH, but also a marker of disease activity and risk of relapse. Aarslev et al. investigated the expression of soluble PD-1 (sPD-1), in 47 healthy controls and 67 AIH patients, 9 with active disease, 31 responders and 27 incomplete-responders to standard treatment (55). sPD-1 is shed from activated T cells and its levels may therefore reflect *in vivo* T-cell activation. In this cross-sectional study, sPD-1 was found to be significantly elevated in AIH patients with active disease and in incomplete responders to standard treatment compared to responders and healthy controls.

### Macrophage Migration Inhibitory Factor (MIF) CD74-Ligand

Macrophage migration inhibitory factor (MIF) is a pro-inflammatory cytokine that mediates the host response to infection and stress by activating innate and adaptive immune pathways and in particular promotes Th1 immunity, which is a key element of AIH pathophysiology (56). Recently, MIF has been associated with a variety of immune-mediated diseases such as rheumatoid arthritis (RA), systemic sclerosis, and inflammatory bowel disease. CD74, the main ligand of MIF, is a transmembrane protein expressed on several cell types including antigen-presenting cells, epithelial cells and stromal cells. MIF and CD74, measured by ELISA in the serum of 165 patients with AIH, PBC, and controls, were found to be significantly higher in patients with AIH than in healthy controls (57). The calculated serum ratio of CD74/MIF was inversely correlated with serum ALT in AIH patients receiving conventional treatment who experienced a relapse, suggesting that the concentration of MIF in relation to its ligand may constitute a potential marker of immunogenic inflammation in the liver in AIH. Two previously identified polymorphisms with clinical significance in the MIF promoter have also been analyzed in DNA samples from more than 500 patients with AIH, PBC, and controls (57). A higher frequency of the pro-inflammatory and high expression−794 CATT7 allele was found in AIH patients compared to PBC ones. Serum concentrations of MIF and its circulating ligand CD74 were also found to be higher in Japanese AIH patients when compared to healthy subjects (58). In this study MIF genotypes were also investigated and then correlated with serum ALT levels and steroid requirements.

In AIH patients with MIF-173 CC/GC genotypes serum ALT levels and steroid requirement were found to be higher than in patients with MIF-173GG. This suggested that the MIF genotype containing the MIF −173C polymorphism could be a marker of disease severity in AIH.

### Soluble CD163

The role of CD163 as a biomarker of AIH was recently investigated. CD163 is the receptor for haptoglobin-hemoglobin complexes and is expressed almost exclusively on macrophages and monocytes (59, 60). Once activated, the receptor sheds and appears in the blood as soluble (s)CD163 therefore constituting a surrogate of macrophage activity. It is elevated in multiple inflammatory disorders including chronic viral hepatitis, acute liver failure and cirrhosis (61).

In 2016 the role of sCD163 in autoimmune liver disorders was first investigated in 121 patients: 101 had AIH type-1; 13 had variant syndromes of AIH type-1; primary biliary cholangitis; 7 of AIH type-1 and primary sclerosing cholangitis (60). sCD163 was significantly elevated in active AIH, normalizing in complete responders receiving prednisone and azathioprine, but remaining high in the incompletely responding cases. sCD163 was positively associated with ALT, IgG and bilirubin values, and negatively with coagulation factors. Furthermore, sCD163 rapidly fell upon treatment in treatment naive patients. If such results are confirmed in future prospective studies, sCD163 could thereby serve as a biomarker of insufficient treatment and prognosis.

### B Cell Activating Factor

B-cell activating factor (BAFF) is a member of the tumor necrosis factor superfamily and it has an important role in the development of B cells. It recently emerged as a regulator of T cell function and appeared to play a central role in the development of autoimmunity (62). When serum BAFF levels were examined in 55 patients with AIH, 14 patients with acute hepatitis, 33 patients with chronic hepatitis C, and 33 healthy controls, they were higher in AIH patients (63). Moreover, there was a positive correlation between BAFF and ALT, total bilirubin, and soluble CD30 in patients with AIH. However, no correlation was found between BAFF and levels of gammaglobulins or ANA titers. Steroid treatment resulted in marked reduction in serum BAFF levels in AIH patients. When patient were treated with steroids, a dramatic decrease in BAFF was seen by 2 weeks of treatment in parallel to ALT serum levels suggesting a correlation with disease activity.

Despite the potentially interesting role of these biomarkers it should again be pointed out how some of these biomarkers have been found to be increased both in multiple autoimmune disorders and other non-autoimmune inflammatory liver conditions, clearly indicating the need for further, and more conclusive studies.

## New Tools for Biological Therapy

During the last two decades, the progressive introduction of engineered, chimeric or humanized, monoclonal antibodies represented a milestone in a new era of the treatment of immune mediated diseases.

They are usually manufactured to aim at one specific branch of the cytokine network and interfere with the signal mediated by the target molecule at various levels.

In some instances, this approach led to modify the natural history of the disease, as for example, TNFα blockers in inflammatory bowel diseases or IL-1 antagonists in RA.

An essential condition to obtain such results is a comprehensive knowledge of the pathogenesis and identification of the up-regulated signaling pathways. Unfortunately, this is not the case for AIH; therefore, the introduction of monoclonal autoantibodies in the treatment of AIH was somewhat empirical, based on analogies with other autoimmune disease, and usually in single cases or small series of patients (Table 1).

**Table 1:** Potential approaches for immunoterapy of AIH patients.

**Mechanism**	**Molecule**	**Experience**
Anti-TNFα antibodies	Infliximab Adalimubab Etanercept	A B B
Anti-CD20/BAFF antibodies	Rituximab Belimubab Ianalumab	A B B^*^
Anti-IL-17 antibodies	Secukinubab Ixekizumab Brodalumab	B B B
Anti-IL-12/IL-23 antibodies	Ustekinumab Briakinumab	B B
Anti-IL-6 antibodies	Tocilizumab	B
Inhibitors of JAK	Tofacitinib Baricitinib	B B
RORγt inhibitors	–	C
Polyclonal Tregs	–	B^*^
CAR TRegs	–	B^**^
Low dose IL-2 administration	IL-2	A^*^
IL-2 modifiers	F5111.2	C
Mesenchymal stem cells	–	B^*^

*A: clinical experience in AIH; B: clinical experience in other autoimmune diseases; C: no clinical experience, only in vitro or animal model data*.

* *clinical trial currently ongoing*.

** *not directly applicable to AIH patients*.

### Targeting the TNFα Pathway

A few arguments link the TNFα signaling pathway to pathogenesis of autoimmune hepatitis, mostly the high levels found in liver and blood of patients compared to controls. Early observations pointed the Adenine/Guanine polymorphism at position 308 of the TNFα gene (64). In Caucasian adults, it was associated with high basal and inducible level of TNFα, with early onset of disease and with poor response to treatment (65). More recently high levels of circulating TNFα were found in pediatric onset disease (66) and a meta-analysis confirmed the role of the TNF-α-308A/G as a risk factor (67).

Infliximab (IFX) is a recombinant humanized chimeric antibody widely used for the treatment of RA, psoriasis, ankylosing spondylitis, ulcerative colitis, and Crohn colitis. The mechanism of action is neutralizing soluble and transmembrane forms of TNFα thus hampering the activation of effector T-cells.

To date, two small series and two case reports describe the outcome of IFX treatment in 24 patients (68–71).

Weiler-Normann et al. described 11 adults with refractory AIH treated with 3 doses of 5 mg/kg IFX as attack therapy and one infusion every 4 to 8 weeks as maintenance therapy according to the clinical course (68). Results were encouraging, 72% (8/11) of patients achieved full biochemical remission, six of them with complete normalization of IgG levels, and most of them could continue therapy for more than 1 year. In the first pediatric report infliximab allowed to achieve a sustained biochemical response while weaning steroids, improving quality of life and allowing deferral of liver transplantation in a 10 years old girl non responsive to high dose steroids, azathioprine, mycophenolate and tacrolimus (70).

A recent study reviewed retrospectively the records of 11 pediatric patients with inflammatory bowel disease (IBD) and autoimmune liver disease (2 AIH and 9 with ASC). All patients received IFX (5 mg/kg) for their IBD and 6 switched to adalimumab due to allergic reaction or non-response. Liver disease improved in 5 patients while in the remaining cases did not show any negative effect (71).

Tolerability of IFX was not homogeneous in this series. More than 50% of adult patients developed infectious complications, a few of which were serious (68). Conversely IFX was very well tolerated in children or young adolescents, only 1 out of 12 patients suffered a CMV reactivation easily treated (71).

**Figure 2:**
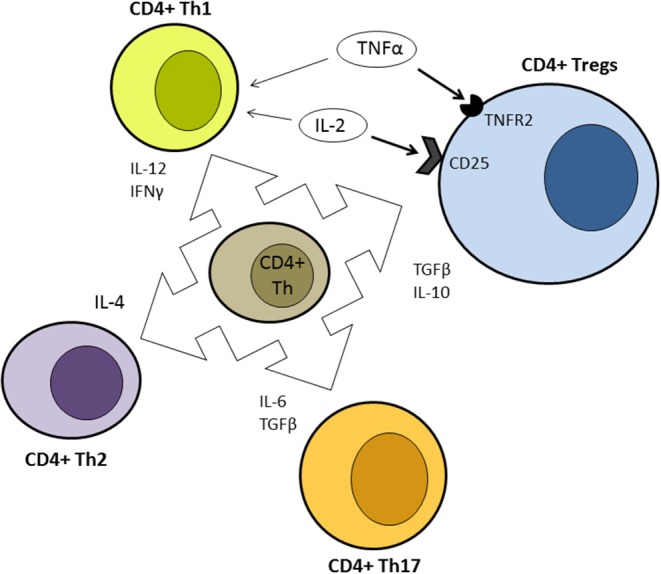
Simplified representation of the differentiation ability of uncommitted CD4+ T helper according to different cytokine stimulation. Contrasting effects of IL-2 and TNFα are highlighted. Bold arrows indicate prevalent effect at low concentration. Th, T helper; Tregs, regulatory T cells.

Besides the infectious risk, the major concern about IFX therapy is the potential risk to trigger other autoimmune diseases (72). This effect descends directly from the complex physiological role of TNFα. The differential activation of the two TNF receptors (TNFR1 and TNFR2) expressed on T cells lead to opposite effects. Stimulation of TNFR1 activate effector T-cells and drives inflammatory response but activation of TNFR2, expressed on regulatory T cells, expands the pool of these cells preventing autoimmunity, (Figure 2) and attenuates inflammation (73). Moreover, TNFα blockade interferes with the normal cytotoxic T lymphocyte suppression of self-reactive B-cell population and with the TNFα-mediated apoptosis of activated T lymphocytes facilitating the expansion of auto-reactive clones (74).

Liver diseases with detection of conventional AIH autoantibodies (ANA, ASMA, and anti-LKM antibody), along with histologic characteristics consistent with autoimmune hepatitis (i.e., interface hepatitis, lymphoplasmacytic infiltrate, and bridging fibrosis) have been reported in dozens of adult patients receiving anti-TNFα therapy to treat various autoimmune diseases. However, it is not clear whether autoimmune features in liver injury during the treatment are primitive or secondary because of the potential role of IFX in causing direct liver damage (75). In every case these features are only anecdotally reported in children and the risk seems marginal (76).

Moreover, stimulation of TNFR2 receptors could be used therapeutically. Vaccination with bacillus of Calmette-Guérin is currently being tested for treatment of various autoimmune diseases and this effect seems to be mediated by the expansion of regulatory T-cells and by selective death of auto reactive lymphocytes caused by activation of TNFα cascade (77).

### Blocking B-Cells Activity

Although pathogenesis of AIH is poorly comprised, a number of observations suggest that the role of T-cells is prominent (78). Analysis of portal lymphoplasmacytic infiltrate in liver biopsies of patient with AIH shows that the large majority of lymphocytes are CD4 T-cells with Th1 functional differentiation (79). The final effectors of liver damage are most likely CD8 T-cells, located at the interface between portal tracts and hepatocytes (80), which are able to cause liver injury directly but also through a bystander effect mediated by soluble cytokines (TNFα, IFNγ) produced by activated T-cells (81). Although hypergammaglobulinemia and the presence of autoantibodies is a hallmark of AIH, it is unlikely that they have a significant pathogenic role. Even if immunoglobulins found on the surface of hepatocytes obtained from AIH liver biopsies could mediate antibody-mediated cell cytotoxicity (82) the passive transfer of serum alone is not able to cause disease in a murine model, as both genetic predisposition and environmental triggers are needed (83). Nevertheless, B-cells likely play a pivotal role in the immunological cascade leading to recognition of auto antigens, mainly acting as APC and thus stimulating activation and proliferation of antigen-experienced T-cells. In AIH type 2 there is a large overlap between the CYP2D6 peptide sequences that are recognized by LKM1 autoantibodies and CYP2D6-specific CD4 cells, which clearly demonstrates the large interplay between B and T cells (84, 85).

Even though the B-depletion strategy in animal models produced contrasting results (86, 87), thanks to the availability of rituximab (RTX), which is well known and widely used in other diseases, this approach has been tested in clinical practice as a rescue therapy.

Rituximab is a chimeric monoclonal antibody that targets the CD20 surface antigen on B-cells causing B-cell depletion (88). It is currently used for treatment of B-cell malignancies such as non-Hodgkin lymphoma and post transplantation lymphoproliferative disease, as well as autoimmune disorders such as RA and ANCA-positive vasculitis. A total of 13 patients who received rituximab as second line therapy for AIH are currently described in literature (89–96). Aside from 4 case reports, only a single study with a small series of adult patient was published. In 2013 Burak et al. reported on 6 patients with incomplete response to conventional therapy receiving 2 doses of 1,000 mg of RTX (89). Transaminases improved in all patients and IgG levels trended downward. Biochemical results were confirmed histologically because at week 48 all patients underwent a control liver biopsy, which showed significantly reduced portal inflammation. A durable weaning of steroid dose was possible in 4 patients, but the only subject who was taken off prednisone at week 24 experienced a relapse at week 46, which temporarily required a higher dose of prednisone to control his disease. Overall, RTX was effective, at least as a steroid sparing agent, but more importantly, it was safe. No adverse reactions were recorded, and at week 72 IgG levels were on the lower side of normal range in all patients; moreover, two infusions did not cause severe hypogammaglobulinemia in adult patients (89).

In regards to pediatric patients, in 2013 D'Agostino et al. reported on 2 children with severe, refractory, type 1 autoimmune hepatitis in which rituximab was administered as rescue treatment allowing to achieve complete clinical and biochemical remission without side effects and without permanent hypogammaglobulinemia (95). A more aggressive protocol was used and patients received a complete cycle of 4 infusions at dose of 375 mg/m^2^ plus a variable number of doses every 4 to 6 months to maintain remission. Prednisone was tapered to a very small dose but suspension was not attempted.

It must be noted that the reported effect of RTX on transaminases parallels the decrease of IgG levels, and it is very slow. Complete remission was achieved from 6 to 12 months later in published series (89, 95) but in some cases it was durable. A good example is the history of a 16 years girl with very active type 1 disease despite triple immunosuppression (prednisone, CSA, and MMF) who received 4 infusions of RTX as described by D'Agostino. After 6 months, no significant effect was noted as regards of transaminase activity, a few weeks later the girl was lost to follow up. We saw the patient again 2 years later with normal transaminases and IgG levels without taking any drug since she had arbitrarily withdrawn all treatments 2 years before (personal unpublished data).

Despite such little amount of published cases, RTX is extensively used as rescue therapy in clinical practice as showed by a recent real life survey among expert centers (97) and a multicentric study not yet published “in extenso” (98). The most dreaded infectious complication of RTX treatment, however, is a progressive, multifocal, and eventually lethal encephalitis caused by the Polyoma John Cunningham virus (JC), which may occur months or years after the beginning of the treatment (99).

A similar but most selective approach, comparing to anti-CD20 antibodies, consists on selective down regulation of the B cell activation factor (BAFF) pathway that is clearly up regulated in AIH patients (63). Biological effects of BAFF comprehend prolonging survival of mature B cells, stimulating IgG production in activated plasmocytes but also stimulating CD4 activated T-cells and probably concurring to differentiation into Th1/Th17 phenotype (62, 100). Blockade of the BAFF signal also increases the number of regulatory B cells (100). A humanized antibody against the soluble BAFF called belimumab was approved for treatment of LES. Another antibody, Ianalumab, is currently evaluated in a controlled randomized trial for second line therapy in AIH (Table 2). Lanalumab target selectively the BR3 receptor of BAFF and act with two distinct mode of action, interfering with the BR3 mediated BAFF signal and causing also antibody-mediated cytotoxicity. Thus, lanalumab administration should deplete preferentially differentiated B cells and plasma blasts sparing pro- and pre-B cells that are instead targeted by anti CD20 antibodies. If the ongoing trial will demonstrate efficacy we could expect a better safety profile than rituximab.

**Table 2:** Selection of ongoing interventional clinical trial from a total of 25 trials registered on Clinicaltrial.gov.

**Title**	**Identifier**	**Status**
Use of erythropoietin to expand regulatory T cells in autoimmune liver disease	NCT03842254	Enrolling by invitation
ADCC mediated B-Cell depletion and BAFF-R blockade	NCT03217422	Recruiting
A single-arm, phase IIa, safety and efficacy trial of selected MSCs in the treatment of patients with PSC & AiH	NCT02997878	Recruiting
Safety and efficacy study of regulatory T cells in treating autoimmune hepatitis	NCT02704338	Unkown status
Liver test study of using JKB-122 in AIH patients	NCT02556372	Active. Not recruiting
sPIF clinical study protocol for autoimmune hepatitis	NCT02239562	Completed. No results
The role of sodium chloride and the Treg/Th17 axis in autoimmune hepatitis	NCT02050646	Recruiting
Induction of regulatory T cells by low dose IL2 in autoimmune and inflammatory diseases	NCT01988506	Recruiting
Umbilical cord mesenchymal stem cells for patients with autoimmune hepatitis	NCT01661842	Unknown status
Effect of high-protein high-fiber diet in patients with autoimmune hepatitis	NCT01655121	Completed. No results

*Selection was done on the basis of the innovative approach to treatment. accessed May 20, 2019*.

### IL-17 and IL-6 Axis: A New Generation of Monoclonal Antibodies

Th17 lymphocytes, a subset of CD4+ cells, are the more recent characterized phenotype after Th1 and Th2 cells (101). They are generated by activated CD4 cells under differentiation signal given by IL6 and TGFβ and may constitute one of the first specialized lineage of T lymphocytes to appear in response of inflammation. Th17 cells secrete mainly IL-17A, IL-17F, IL-21, IL-22 but also IL-6 that, along with IL-23, is crucial for survival and expansion of Th17 cells (102) (Figure 2). As the majority of components of adaptive immune system, Th17 cells have proinflammatory and cytotoxic effect but also a regulatory potential to avoid excessive inflammatory response. TGFβ and IL-2 are negative signals for differentiation and amplification of Th17 cells (103) and IL-22 have anti-inflammatory effect and promote damage repair and tissue regeneration (104).

The positive signal is mediated mainly by IL-17 and IL-21 that induce a broad reaction of different cell types to secrete cytokines (IL-1, IL-6, TNFα, IL-8, GM-CSF, G-CSF), chemokines (CXCL1, CXCL10), and metalloproteinases and induce recruitment, activation and migration of Th1 cells and neutrophils (105). IL-2 and IL-17, result of synergistic action of Th1 and Th17 cells, induce hepatocytes to produce IL-6 and to express class II MHC molecules that boost Th17 cells differentiation and CD8 cells activation (106, 107). IL-21 together with TGF-β further amplifies Th17 differentiation in an autocrine self-amplification loop.

Patients with AIH show signs of up regulation of IL-17 pathway: they have increased IL-17 blood and liver concentration and more Th17 infiltrating cells than healthy and other chronic liver disease controls (106, 108).

Intrahepatic production of IL-17 and other related mediators as IL-6 and IL-23 were associated with disease activity and liver fibrosis (108).

In summary, Th17 cells are pivotal players of early phase of inflammatory response, probably also in AIH. IL-1, IL-6, IL-17, and IL-21 axis are essential to trigger and maintain Th17 response and consequently they are potential therapeutic targets for a new generation of monoclonal antibodies (108) (Figure 2). In animal models Il-17 inhibition was able to improve AIH disease activity, moreover anti-IL-17 antibodies increased regulatory T-cells (Tregs) production and expansion from sera of AIH patients (109).

Several antibodies that interfere with soluble IL-17, such as secukinubab and ixekizumab, or with IL-17 receptor, such as brodalumab are being tested for various autoimmune diseases (Table 1). Published Phase II, randomized, double blind, placebo-controlled studies using these three humanized monoclonal antibodies showed positive results in treatment of Psoriasis. Secukinubab was superior to anti-TNFα in therapy of Psoriasis showing better control of disease and longer lasting effects with a good safety profile as shown by results from the phase 3 FUTURE 2 study and confirmed by data from real word US registry (110, 111). Positive results were also described in the clinical trial of secukinumab and ixekizumab in various IL-17- related immune mediated disease, such as Rheumatoid Arthritis (RA), ankylosing spondylitis, and non-infectious uveitis (112–114).

Even promising these results cannot be transposed in therapy of autoimmune hepatitis and pilot clinical studies are needed to confirm preliminary data although the unfavorable outcome of secukinumab for the treatment of Crohn's disease induced caution in clinicians and pharmaceutical companies (115).

Another potential approach to inhibit the IL-17 axis is to target cytokines that are essential for differentiation and survival of Th17 cells as IL-1β, IL-6 and IL-23, as mentioned above (108).

IL-12 and IL-23 share the same p40 subunit that is targeted by specific humanized antibodies such as ustekinumab and briakinumab. Both were demonstrated effective and safe in Phase II/III clinical trials for the treatment of active psoriatic arthritis, plaque psoriasis or refractory Crohn's disease (116, 117) but only ustekinumab was approved by regulatory authorities and is currently available for use in treatment. Ustekinumab showed very good long-term safety in moderate-to-severe psoriasis after up to 5 years of follow-up, and superiority to etanercept in a large Phase III trial (118). Preliminary favorable data exist with regards to Behçet's disease (119) but it was not effective in trials involving patients with multiple sclerosis and primary biliary cholangitis (120, 121). Moreover, antibodies against the IL-23 unique subunit p19 are currently tested in clinical trials with the hope to obtain a more selective action and a better safety profile (122).

Also IL-6, which is essential to induce selection of CD4+ naive lymphocytes toward Th17 differentiation, became a target for immunotherapy. Tocilizumab, a humanized antibody against IL-6 receptor has been approved for therapy of RA, juvenile idiopathic arthritis and giant cell arteritis (123–125) and the field of application is expanding toward other vasculitis and even severe immunoallergic diseases.

The most recent approach for immunotherapy, rather than interfering with a single cytokine, is blocking the intracellular common downstream pathways that react to the interaction between cytokines and their receptors. Potential targets in such perspective are cytoplasmic signaling protein and nuclear transcription factors and drugs are not antibodies but small molecules that are able to cross cellular membranes.

Type I and type II cytokine receptors are a super-family of receptors that recognize over 50 cytokines, interleukins and interferons, including IL-2, IL-4, IL-6, IL-21, all involved at various titer in AIH pathogenesis. Al members of this family of receptors use Janus kinases (JAK) 1–3 and tyrosine kinases 2 to transfer the input to downstream signal transducers. Consequently, inhibitors of JAK signaling (jakitinibs), have the potentiality to hamper the differentiation in Th17 cells, mainly blocking the IL-6 stimulation (126). Tofacitinib and baricitinib, are small molecules that interfere with the ATP binding domains of kinases, and have been approved for RA and ulcerative colitis treatment. More importantly, tofacitinib proved successful in treating patients with RA where antibodies against single cytokines or single cytokine receptors, such as TNFα and IL-6 (126), were ineffective.

As regards transcription factors, the two key molecules involved in Th17 differentiation are the signal transducer and activator of transcription (STAT) 3 proteins and orphan nuclear receptor RORγ. The specific isoform RORγt was the first transcription factor found to be selectively expressed in TH17 cells and is regulated by STAT3. More recently also RORα was identified as synergistic of the RORγt isoform (127). Being the master regulator for Th17 differentiation RORγt has the potential to be a promising therapeutic target for autoimmune disease (128).

Development of RORγt specific inhibitors is one of the most promising fields of research for autoimmune diseases. Moreover, this class of drugs might have a more selective action and consequently better safety profile when compared to kinases inhibitors, which are potent immunosuppressive drugs with relevant infectious complications.

## Regolatory T-Cells: Cutting the Roots of Disease

Negative selection of self-reactive T cells in the thymus is the first line of defense against autoimmunity and is responsible for neutralization of most high-affinity T cell recognizing self-antigens. Nevertheless, accurate selection and/or modulation of T cell activation in peripheral tissues is pivotal to avoid inappropriate reaction against targets as food antigens, commensal microorganisms, environmental antigens, or simply to avoid excessive damage caused by inflammatory response. A heterogeneous subset of T cells has the ability to suppress the function of other lymphocytes and it consists of various populations: CD4^+^CD25^+^Foxp3^+^ cells, IL-10-producing CD4^+^ Tr1 cells, TGFβ-producing Th3 cells, CD8^+^ cells, NK T cells, CD4–CD8^−^ T cells, and γδ T cells. Among them, regulatory T cells, or Tregs, identified by the expression of CD4, CD25, and Foxp3 are the most studied and are probably the major players in the maintenance of immune tolerance and homeostasis (129, 130).

Two main subtypes of Tregs have been described. Natural Tregs generated in the thymus, constitute 5–10 % of the CD4^+^ lymphocytes in the peripheral blood (131). Induced regulatory T cells develop in periphery after antigen exposure and stimulation by appropriate signals as TGF-β (132). Both lineages share similar functional features. Tregs suppress immune-mediated responses by several mechanisms: they inhibit production of IFNγ and IL-17, produce IL-10 to enforce the anti-inflammatory cytokine pathway (133, 134), induce production of small anti-inflammatory molecules like adenosine (135) and directly interact with and induce apoptosis of Th1 cells via the galectin-9-TIM-3 pathways (136). Moreover, Tregs have been described as capable of modulating other cells of the adaptive immune system, including CD8^+^ T cells and B cells, and able to interact with many components of the innate immune system, such as dendritic cells, macrophages, neutrophils, and γδ T cells (137).

In human pathology, genetically determined severe loss of function of Tregs causes multiorgan autoimmune disease such as the autoimmune polyendocrinopathy-candidiasis-ectodermal dystrophy (APECED) caused by mutations of the gene AIRE (138) or the immunodysregulation, polyendocrinopathy, enteropathy, X-linked (IPEX) syndrome, a catastrophic and often lethal systemic disease secondary to critical dysfunction of Foxp3 (139).

A putative role of impaired T-cells regulatory function in the pathogenesis of multifactorial autoimmune diseases was extensively investigated, but is still debated. Tregs are a very heterogeneous population and are able to change phenotype according to the local cytokine environment (140). Moreover, the number of circulating Tregs is highly variable during an inflammatory process, they move to the target organ and their blood count decreases as demonstrated during acute rejection in transplanted patients (141).

In autoimmune hepatitis Tregs function was accurately investigated, both in adults than in pediatric patients, by the group of the King's College. In the early studies a numerical and functional deficiency of Tregs was suggested; they were decreased in number and with defective expansion under stimulation (142), the expression of Foxp3 was lower, co-culture with effectors cells lead to decreased TGFβ and increased IL-4 secretion and spontaneous apoptosis of CD4^+^ CD25^−^ was defective in pediatric patients (143). In adult patients the defect in immunoregulation was more complex involving Tregs but also other regulatory cell populations (144). The more recent studies focused on CD39^+^ Tregs producing adenosine and found multiple defects, both in their numbers and functions (145). Moreover, Tregs showed a decreased capability to produce IL-10 and suppress target cell proliferation (146). Despite these results, recent studies on adult patients with AIH failed to confirm a functional defect in Tregs compartment. They were numerically comparable to healthy subjects in both blood and liver, and exhaustive *in vitro* functional studies were fully normal (147, 148). In any case, the possibility to treat AIH potentiating the immunoregulatory properties of endogenous regulatory cells generated great enthusiasm because of the potential ultra-selective action with virtual absence of adverse effects and as it offers the chance to achieve long-term tolerance to liver autoantigens. Many approaches are theoretically possible.

### *Ex vivo* Expanded Tregs. Adoptive Immunotherapy

Autologous *ex vivo* expanded Tregs could represent an elegant approach but a few issues make this approach a tough challenge. First, Tregs are activated via the TCR receptor and, as effector cells, are peptide-specific. This means that pathogenic autoantigens must be known and available for manipulation. Second, infused cells must find the way to correctly localize into the target organ as they act with short-range mechanisms (direct interaction or paracrine signals). Third and lastly, manipulation of these cells must take place according to strict good manufacturing practices (GMP) to ensure high purity of the product. This is very expensive and requires a complex organization, and availability of GMP-compliant reagents or intermediate products could restrict the feasibility of innovative techniques.

Tregs can be isolated from circulating mononuclear cells (PBMCs) according to the most appropriate markers set, in most recent studies CD4^+^/CD25^high^/CD127^−/low^, then activated and expanded by non-specific antigen stimulation, usually co-activation of CD3 and CD28 in presence of IL-2. This method leads to a polyclonal Tregs population (149). In the animal model cited above (15) adoptive transfer of *ex vivo* expanded Tregs in mice with AIH restored peripheral tolerance to the target antigen formimidoyltransferase cyclodeaminase and induced remission of AIH (150). In this model CD4^+^CD25^+^FoxP3^+^ Tregs from xenoimmunized mice expressed high levels of the liver-homing chemokine receptor CXCR3, the cognate receptor of CXCL9 and CXCL9 (151), allowing a correct localization of Treg into the target organ.

Although polyclonal Tregs are less effective in controlling autoimmune responses than monoclonal antigen-specific Tregs, little clinical experience exists. The only concluded clinical study in liver pathology involved 10 adult liver-transplanted patients. A co-culture of donor with recipient lymphocytes in the presence of co-stimulatory signals generated a population enriched with polyclonal Tregs, partially matched with donor antigens. Administration of this product allowed tapering followed by discontinuation of immunosuppressive agents within 18 months in 7 out of 10 patients (152). A Chinese phase 1 study using polyclonally expanded Tregs is ongoing in AIH patients resistant to conventional treatment (Table 2). Besides liver diseases, polyclonal Tregs HLA-matched or partially matched were clinically tested to treat graft vs. host disease in allogenic stem cell transplantation with some positive effects (153–155) and in therapy of Type 1 diabetes both in adults and in children (156, 157). These results encouraged the research for more specific Tregs populations. A proof of concept that autoantigens-specific Tregs are able to revert established autoimmune diseases was obtained in animal model of type 1 diabetes (158, 159) but also in humans antigen-specific regulatory T cells generated by T cell receptor gene transfer can be obtained (160).

A promising simple methodology to obtain a therapeutic product with high percentage of autoantigen-specific Tregs is based on surface markers such as latency-associated peptide (LAP) and glycoprotein A repetitions predominant (GARP) that are expressed by stimulated Tregs but not by effectors Tcells which conversely express CD154. Selection and expansion of CD4+ CD25+ CD127-/low GARP+/LAP+ CD154- cells increases the Tregs cell purity to over 90%, most of them already activated against the ongoing autoimmune disease (161).

In transplantation medicine various methods have been developed to obtain the so-called donor alloantigen reactive Tregs (darTregs), a mixture of polyclonal recipient derived Tregs enriched in cells activated by donor alloantigens (162, 163). DarTregs are currently evaluated in several phase I or II trials but unfortunately this concept can't be exported in the setting of autoimmune diseases.

The technology of chimeric antigen receptor (CAR) T cells, developed in oncology, might theoretically be adapted to potentiate an antigen-specific regulatory effect. This methodology consists in transducing T cells with a chimeric protein made with an extracellular antigen binding domains, properly selected, joined to transmembrane and intracellular co-stimulatory domains. Activation of CAR by the antigen causes an intracellular signal that overrides the original signaling via the TCR. In oncology CARs recognizing tumor antigens are transduced into autologous effector Tcells (164) but CARs can also be transduced in T cells with regulatory phenotype that, once expanded, represents a monoclonal antigen specific Tregs population (165). CAR Tregs against donor HLA were more effective than polyclonal Tregs to improve rejection in a human skin xenograft transplant model (166). The production of monoclonal liver-specific CARs, however, requires the knowledge of pathogenetic antigens, which is the case for type 2 AIH but not for type 1 AIH or PSC.

A different approach to generate T-regs specific for CYP2D6 immunodominant regions was described in 2011 by Longhi et al. (167). In this model Tregs isolated from patients carriers of predisposing HLA-DR7 and/or HLA-DR3 alleles were co-cultured with CYP2D6-peptide-loaded semi-mature DCs. The final product, an oligoclonal mixture of Tregs reacting against CYP2D6 epitopes, was able to suppress *in vitro* expansion of effector cells (167). Although promising, these results did not lead to application in clinical trials.

### *In vivo* Stimulation of Tregs: The IL-2 Pathway

IL-2 is a key factor of the immune system cytokine network. IL-2 is essential to promote response of effector cells but also expansion, survival, and maintenance of regulatory function of Tregs. This dual effect is mediated by the complex structure of the receptor (IL-2R). The high affinity trimeric form is composed of the subunit IL-2Rα (CD25) and the subunits IL-2Rβ and IL-2Rγ. Both Tregs and activated CD4 cells may express this trimeric form (Figure 2) while CD8 and NK cells bear low affinity dimeric form that lacks CD25 (168). IL2RA deficient mice shows a complete deficiency of regulatory T cells and develop multiorgan lethal autoimmune disease involving skin, intestine, lung, and liver (169). In mature immune systems, however, effects depend on IL-2 local concentration: exogenous IL-2 typically amplifies effector T cells response but preferential activation of high affinity receptor by low dose IL-2 determine a prevalent suppressor effect (168). A few clues suggest an association between IL-2 signal deregulation and autoimmune liver diseases. GWAS studies indicated an association of polymorphism of IL-2 and IL2Ra with PBC and PSC but not with AIH (170). Liver-derived lymphocytes from patients with PSC show reduced expression of the IL-2 receptor (171). In AIH patients, baseline and under therapy IL-2 serum levels seems lower than controls (172) and children with higher baseline levels of IL-2 more easily achieve biochemical remission (173).

Low dose IL-2 therapy could be a good tool to potentiate *in vivo* Tregs pool and to stimulate suppressor function. Clinical studies have already shown positive effects in patients with different autoimmune disease including SLE and diabetes (174, 175) and an ongoing trial is enrolling patients with AIH (Table 2) despite the finding of a possible defective regulatory T-cell responsiveness to IL-2 (146). To date only two patients with AIH have been treated with low dose IL-2 as rescue therapy: even if only one patient reached remission, in both blood Tregs number increased after therapy (176). Although *in vitro* studies of liver explants showed that low dose IL-2 increases activity of Tregs but not of Teff, clinical response is not completely predictable, and a worsening of disease has been observed in patients with diabetes (177). For these reasons another attractive approach to up regulate *in vivo* Tregs function through IL-2 stimulation is currently under investigation. IL-2 is a molecule with high conformational flexibility; this property can be used to selectively target Tregs. A recently developed fully human antibody stabilizes IL-2 in a conformation that preferentially potentiates Tregs activity. In a mouse model of autoimmune encephalomyelitis it reduced disease severity and induced remission of type 1 diabetes in the NOD mouse (178).

## Other Therapeutic Options

Mesenchymal stem cells (MSC) are a lineage of multipotent cells that can differentiate in all mesenchymal-derived cells as osteoblasts or adipocytes. They can be isolated from bone marrow, umbilical cord blood but also by peripheral tissue. MSC suppress differentiation of naïve T Cells into effector cells (Th1, Th17) promoting maturation toward Treg phenotype. They counteract proliferation of NK and CD8^+^ cells and they induce tolerogenic phenotype in dendritic cells. This action is mainly mediated by humoral signals such as TGFβ, IL-10, nitric oxide and many others and are not dependent from direct contact with target cells, thus a correct homing of these cells is less important (179).

First implementation of MSC therapy in autoimmune liver disease involved patients with PBC. In two separate pilot studies a total of 17 patients were treated with positive results, their quality of life substantially improved. More importantly, the safety profile was excellent (180, 181). Two studies on large cohorts of AIH patients are currently ongoing (Table 2).

Erythropoietin is a well-known molecule produced by the kidney in response to hypoxia that stimulates bone marrow to produce red blood cells. Recently it was recognized to have immune-modulating and tissue-protective effects. CD4+ and CD8+ T cells express EPO receptor and experimental data showed that EPO has a direct inhibitory effect on effector/memory T cells, while it promotes formation of regulatory T cells (182). A pre-phase 1 study is ongoing to evaluate change of Tregs number and cytokines profile in the blood of AIH patients after a single administration of EPO (Table 2).

## Intestinal Microbiome and Dietary Interventions

The growing knowledge about the role of intestinal microbiome in almost every human physiological pathway is probably the most relevant advance in medical research in recent years. The human gut hosts 10–11 trillion bacteria comprising 500–1500 different species from four major phyla: Actinobacteria, Firmicutes, Proteobacteria, and Bacteroidetes (183). The interplay between intestinal innate and adaptive immune system and bacteria can influence systemic immune response, trigger autoimmunity or determine tolerance against bacterial or alimentary xenoantigens. Main factors deciding the outcome of this interaction are intestinal barrier permeability, genetic host variability and composition of the microbiome (184). Activated lymphocytes against products that mimic normal self-proteins, bacterial antigens or simply cytokines (intestinal inflammasome) generated in the intestinal tract may translocate in systemic circulation and produce peripheral immune-mediated tissue damage (185). Variation of the composition of the microbiome has been associated with several systemic immune-mediated diseases, including type 1 diabetes (186), inflammatory bowel disease (187) and rheumatoid arthritis (188) but also with PSC and AIH (189, 190). In a novel mouse model of AIH, active disease was associated with reduced diversity and total load of intestinal bacteria (191). Liver-infiltrating lymphocytes in PSC include A4β7^+^CCR9^+^CD8^+^ memory T cells activated in the gut and recruited to the liver by aberrant expression of the gut-specific chemokine CCL25 on the hepatic endothelium (192). Microbiome composition may also confer protection against autoimmune diseases and this property is transmissible to other individuals. It is found to differ in Male and female NOD mice, and this characteristic is lost after male castration. Female NOD mice are more prone to develop type 1 diabetes than male. Colonization of immature female NOD mice with the intestinal microflora from mature male NOD mice protects from diabetes (193). So far incomplete comprehension of multiple factors affecting the microflora composition do not allow translation to clinical practice, but a huge amount of data have already been accumulated, for instance, by the Humane Microbiome Project, and initial practical results are conceivable in a near future. Specific aspects, however, are currently being investigated. Toll-like receptor 4 (TLR4) is a recognition receptor for bacterial peptides expressed on hepatocytes, liver sinusoidal endothelial cells, Kupffer cells and hepatic stellate cells. As the other toll-receptor family members expressed by enterocytes, according to quality and intensity of stimulating bacterial components, it can induce tolerance or promote immune activation and liver fibrosis. JKB-122 is a TLR4 antagonist that demonstrated ability to reduce hepatocyte necrosis and potentiate effect of low dose prednisolone in an experimental mouse model for liver injury (194). A pilot clinical trial is currently underway (Table 2).

Microflora composition is influenced by a number of variables, among that alimentary behavior is the most easy to manipulate. High sodium diet has detrimental effects on immunological homeostasis by several mechanisms. Indirect effects are mediated by the change of composition of microbiome, higher sodium concentration decrease number of Lactobacillus spp that have the property of hamper Th17 polarization trough the increase of indole production, a metabolite of tryptophan (195). On the other side, high sodium concentration enhances Th17 differentiation trough the induction of osmosensitive nuclear factor of activated T cells (NFAT) 5 that finally leads to potentiate activity of IL-23 receptor and of the mTOR pathway (196). A trial on the effects of low sodium diet is currently recruiting patients with AIH (Table 2). Another study on the effects of a high fiber, high protein diet in AIH patients was recently concluded but results are pending. The rationale of this trial is that dietary fibers are fermented by gut microbiota to short-chain fatty acids (SCFA) as propionate, acetate or butyrate. SCFA exert multiple regulatory activities, increase colonic Tregs number, but also increase FOXP3 expression and secretion of anti-inflammatory IL-10 (197). This diet already showed positive effects on a mouse model of AIH in terms of disease activity, higher Treg/Th17 cell ratio and decrease of intestinal permeability (198). Enhanced intestinal permeability with elevated blood concentrations of lipopolysaccharides was found in a population of Chinese patients with AIH (199).

## Conclusions

Autoimmune hepatitis is a complex disease. The clinical onset can be widely varied, and ranges between hyper-acute hepatitis with liver failure to chronic, unapparent disease with mild, fluctuating increase of liver enzymes, the latter usually leading to a late diagnosis with severe, established fibrosis. Besides typical cases with prominent hypergammaglobulinemia and high-titer autoantibodies, diagnosis is usually challenging: autoantibodies may be absent in up to 20% of cases and liver histology may show inconclusive pictures, overlapping with features of bystander hepatitis, which is very common in several systemic diseases. The scoring systems proposed so far are more useful for research purposes than for clinical use, especially in children. Once diagnosis is made, the short-term management is often simple. Currently available treatments induce remission in almost the totality of cases and clinicians might have the erroneous feeling that the patient is cured. Indeed, the long-term management of the disease is the most challenging task. Once biochemical remission is achieved, there are no reliable biomarkers of underlying activity to guide the tapering of immunosuppression and to estimate the risk of relapse at suspension of therapy. Current available drugs are able to avoid further liver damage but do not cure the imbalance of the immune system that is the root of the disease. A few patients achieve spontaneous desensitization against liver antigens during conventional therapy and do not relapse at suspension but the large majority of them experiences an extenuating sequence of relapses and often serious adverse effects of steroid therapy that finally compromise the compliance, especially in female adolescents. The worse situation to face is an uncontrolled disease in an adolescent with a long history of disease and multiple relapses with no second line treatment remaining to propose.

The ideal therapy should be enough selective to contrast immunomediated liver damage while preserving or potentiating the ability to develop permanent tolerance vs. pathogenic autoantigens. Indeed, the inadequate knowledge of the autoantigens involved in the pathogenesis of AIH constitutes a major obstacle for research. Recent progress in molecular biology, however, made available a number of potential new therapeutic strategies, some of them already tested in small group of patients or, more frequently, in other autoimmune disease (Table 1). Future advancements depend on the availability of large registries of patients that may be the basis for international collaborative clinical trials.

## Author Contributions

MS: conceptualization, writing—original draft. SN and GM: validation, writing—review, and editing.

### Conflict of Interest Statement

The authors declare that the research was conducted in the absence of any commercial or financial relationships that could be construed as a potential conflict of interest.

## References

[1] Maggiore G Nastasio S Sciveres M. Juvenile autoimmune hepatitis: spectrum of the disease. World J Hepatol. (2014) 6:464–76. 10.4254/wjh.v6.i7.464 25067998 PMC4110538

[2] Gregorio GV Portmann B Reid F Donaldson PT Doherty DG McCartney M. Autoimmune hepatitis in childhood: a 20-year experience. Hepatology. (1997) 25:541–7. 10.1002/hep.510250308 9049195

[3] Deneau M Jensen MK Holmen J Williams MS Book LS Guthery SL. Primary sclerosing cholangitis, autoimmune hepatitis and overlap in Utah children: epidemiology and natural history. Hepatology. (2013) 58:1392–400. 10.1002/hep.26454 23686586

[4] Maggiore G Bernard O Homberg JC Hadchouel M Alvarez F Hadchouel P.. Liver disease associated with anti-liver-kidney microsome antibody in children. J Pediatr. (1986) 108:399–404. 10.1016/S0022-3476(86)80880-0 3950819

[5] Maggiore G Veber F Bernard O Hadchouel M Homberg JC Alvarez F.. Autoimmune hepatitis associated with anti-actin antibodies in children and adolescents. J Pediatr Gastroenterol Nutr. (1993) 17:376–81. 10.1097/00005176-199311000-00007 8145091

[6] Maggiore G Porta G Bernard O Hadchouel M Alvarez F Homberg JC.. Autoimmune hepatitis with initial presentation as acute hepatic failure in young children. J Pediatr. (1990) 116:280–2. 10.1016/S0022-3476(05)82892-6 2299503

[7] Caprai S Vajro P Ventura A Sciveres M Maggiore G SIGENP Study Group for Autoimmune Liver Disorders in Celiac Disease. Autoimmune liver disease associated with celiac disease in childhood: a multicenter study. Clin Gastroenterol Hepatol. (2008) 6:803–6. 10.1016/j.cgh.2007.12.002 18258488

[8] Routes JM Verbsky JW. Immunodeficiency presenting as an undiagnosed disease. Pediatr Clin North Am. (2017) 64:27–37. 10.1016/j.pcl.2016.08.007 27894450

[9] Mieli-Vergani G Vergani D Baumann U Czubkowski P Debray D Dezsofi A.. Diagnosis and management of pediatric autoimmune liver disease: ESPGHAN hepatology committee position statement. J Pediatr Gastroenterol Nutr. (2018) 66:345–60. 10.1097/MPG.0000000000001801 29356770

[10] Vergani D Alvarez F Bianchi FB Cançado EL Mackay IR Manns MP.. Liver autoimmune serology: a consensus statement from the committee for autoimmune serology of the International Autoimmune Hepatitis Group. J Hepatol. (2004) 41:677–83. 10.1016/j.jhep.2004.08.002 15464251

[11] Bridoux-Henno L Maggiore G Johanet C Fabre M Vajro P Dommergues JP.. Features and outcome of autoimmune hepatitis type 2 presenting with isolated positivity for anti-liver cytosol antibody. Clin Gastroenterol Hepatol. (2004) 2:825–30. 10.1016/S1542-3565(04)00354-4 15354284

[12] Gueguen M Meunier-Rotival M Bernard O Alvarez F. Anti-liver kidney microsome antibody recognizes a cytochrome P450 from the IID subfamily. J Exp Med. (1988) 168:801–6. 10.1084/jem.168.2.801 2842431 PMC2189003

[13] Lapierre P Hajoui O Homberg JC Alvarez F. Formiminotransferase cyclodeaminase is an organ-specific autoantigen recognized by sera of patients with autoimmune hepatitis. Gastroenterology. (1999) 116:643–9. 10.1016/S0016-5085(99)70186-1 10029623

[14] Muratori L Parola M Ripalti A Robino G Muratori P Bellomo G.. Liver/kidney microsomial antibody type 1 targets CYP2D6 on hepatocyte plasma membrane. Gut. (2000) 46:553–61. 10.1136/gut.46.4.553 10716687 PMC1727874

[15] Lapierre P Djilali-Saiah I Vitozzi S Alvarez F. A murine model of type 2 autoimmune hepatitis: xenoimmunization with human antigens. Hepatology. (2004) 39:1066–74. 10.1002/hep.20109 15057911

[16] Maggiore G Socie G Sciveres M Roque-Afonso AM Nastasio S Johanet C.. Seronegative autoimmune hepatitis in children: spectrum of disorders. Dig Liver Dis. (2016) 48:785–91. 10.1016/j.dld.2016.03.015 27079745

[17] Alvarez F Berg PA Bianchi FB Bianchi L Burroughs AK Cancado EL International Autoimmune Hepatitis Group Report: review of criteria for diagnosis of autoimmune hepatitis. J Hepatol. (1999) 131:929–38. 10.1016/s0168-8278(99)80297-9 10580593

[18] Ebbeson RL Schreiber RA. Diagnosing autoimmune hepatitis in children: is the International Autoimmune Hepatitis Group scoring system useful? Clin Gastroenterol Hepatol. (2004) 2:935–40. 10.1016/S1542-3565(04)00396-9 15476158

[19] Hennes EM Zeniya M Czaja AJ Parés A Dalekos GN Krawitt EL.. Simplified criteria for the diagnosis of autoimmune hepatitis. Hepatology. (2008) 48:169–76. 10.1002/hep.22322 18537184

[20] Hiejima E Komatsu H Sogo T Inui A Fujisawa T. Utility of simplified criteria for the diagnosis of autoimmune hepatitis in children. J Pediatr Gastroenterol Nutr. (2011) 52:470–3. 10.1097/MPG.0b013e3181fc1e0b 21407112

[21] Miao Q Bian Z Tang R Zhang H Wang Q Huang S.. Emperipolesis mediated by CD8 T cells is a characteristic histopathologic feature of autoimmune hepatitis. Clin Rev Allergy Immunol. (2015) 48:226–35. 10.1007/s12016-014-8432-0 25051956

[22] Murray-Lyon IM Stern RB Williams R. Controlled trial of prednisone and azathioprine in active chronic hepatitis. Lancet. (1973) 1:735–7. 10.1016/S0140-6736(73)92125-9 4121073

[23] Maggiore G Bernard O Hadchouel M Hadchouel P Odievre M Alagille D. Treatment of autoimmune chronic active hepatitis in childhood. J Pediatr. (1984) 104:839–44. 10.1016/S0022-3476(84)80477-1 6726513

[24] Vegnente A Larcher VF Mowat AP Portmann B Williams R. Duration of chronic active hepatitis and the development of cirrhosis. Arch Dis Child. (1984) 59:330–5. 10.1136/adc.59.4.330 6721559 PMC1628653

[25] Mieli-Vergani G Vergani D. Autoimmune paediatric liver disease. World J Gastroenterol. (2008) 14:3360–7. 10.3748/wjg.14.3360 18528933 PMC2716590

[26] Vitfell-Pedersen J Jørgensen MH Müller K Heilmann C. Autoimmune hepatitis in children in Eastern Denmark. J Pediatr Gastroenterol Nutr. (2012) 55:376–9. 10.1097/MPG.0b013e3182602b20 22644464

[27] Cuarterolo ML Ciocca ME López SI de Dávila MT Alvarez F. Immunosuppressive therapy allows recovery from liver failure in children with autoimmune hepatitis. Clin Gastroenterol Hepatol. (2011) 9:145–9. 10.1016/j.cgh.2010.10.013 21029789

[28] Kerkar CN Annunziato RA Foley L Schmeidler J Rumbo C Emre S. Prospective analysis of nonadherence in autoimmune hepatitis: a common problem. J Pediatr Gastroenterol Nutr. (2006) 43:629–34. 10.1097/01.mpg.0000239735.87111.ba 17130740

[29] Alvarez F Ciocca M Canero-Velasco C Ramonet M de Davila MT Cuarterolo M. Short-term cyclosporine induces a remission of autoimmune hepatitis in children. J Hepatol. (1999) 30:222–7. 10.1016/S0168-8278(99)80065-8 10068099

[30] Debray D Maggiore G Girardet JP Mallet E Bernard O. Efficacy of cyclosporin A in children with type 2 autoimmune hepatitis. J Pediatr. (1999) 135:111–4. 10.1016/S0022-3476(99)70339-2 10393616

[31] Hyams JS Ballow M Leichtner AM. Cyclosporine treatment of autoimmune chronic active hepatitis. Gastroenterology. (1987) 93:890–3. 10.1016/0016-5085(87)90454-9 2887482

[32] Czaja AJ. Diagnosis and management of autoimmune hepatitis: current status and future directions. Gut Liver. (2016) 10:177–203. 10.5009/gnl15352 26934884 PMC4780448

[33] Cuarterolo M Ciocca M Velasco CC Ramonet M González T López S.. Follow-up of children with autoimmune hepatitis treated with cyclosporine. J Pediatr Gastroenterol Nutr. (2006) 43:635–9. 10.1097/01.mpg.0000235975.75120.38 17130741

[34] Sciveres M Caprai S Palla G Ughi C Maggiore G. Effectiveness and safety of ciclosporin as therapy for autoimmune diseases of the liver in children and adolescents. Aliment Pharmacol Ther. (2004) 19:209–17. 10.1046/j.1365-2036.2003.01754.x 14723612

[35] Nastasio S Sciveres M Matarazzo L Malaventura C Cirillo F Riva S. Long-term follow-up of children and young adults with autoimmune hepatitis treated with cyclosporine. Dig Liver Dis. (2018) 51:712–8. 10.1016/j.dld.2018.10.018 30502231

[36] Zizzo AN Valentino PL Shah PS Kamath BM. Second-line agents in pediatric patients with autoimmune hepatitis: a systematic review and meta-analysis. J Pediatr Gastroenterol Nutr. (2017) 65:6–15. 10.1097/MPG.0000000000001530 28644343

[37] Nastasio S Sciveres M Matarazzo L Maggiore G. Old and new treatments for pediatric autoimmune hepatitis. Curr Pediatr Rev. (2018) 14:187–95. 10.2174/1573396314666180516130314 29766815

[38] Woynarowski M Nemeth A Baruch Y Koletzko S Melter M Rodeck B.. Budesonide versus prednisone with azathioprine for the treatment of autoimmune hepatitis in children and adolescents. J Pediatr. (2013) 163:1347–53. 10.1016/j.jpeds.2013.05.042 23810723

[39] Mieli-Vergani G Vergani D. Budesonide for juvenile autoimmune hepatitis? Not yet J Pediatr. (2015) 163:1246–8. 10.1016/j.jpeds.2013.06.064 23932214

[40] Ytting H Larsen FS Everolimus treatment for patients with autoimmune hepatitis and poor response to standard therapy and drug alternatives in use, Scand. J. Gastroenterol. (2015) 50:1025–31. 10.3109/00365521.2014.998271 25862144

[41] Chatrath HL Allen TD Boyer Use of sirolimus in the treatment of refractory autoimmune hepatitis. Am J Med. (2014) 127:1128–31. 10.1016/j.amjmed.2014.06.016 24979741

[42] Kumari N Kathuria R Srivastav A Krishnani N Poddar U Yachha SK Significance of histopathological features in differentiating autoimmune liver disease from non-autoimmune chronic liver disease in children. Eur J Gastroenterol Hepatol. (2013) 25:333–7. 10.1097/MEG.0b013e32835a68a1 23085577

[43] Czaja AJ. Rapidity of treatment response and outcome in type 1 autoimmune hepatitis. J Hepatol. (2009) 51:161–7. 10.1016/j.jhep.2009.02.026 19446908

[44] Czaja AJ Menon KV Carpenter HA. Sustained remission after corticosteroid therapy for type 1 autoimmune hepatitis: a retrospective analysis. Hepatology. (2002) 35:890–7. 10.1053/jhep.2002.32485 11915036

[45] Montano-Loza AJ Carpenter HA Czaja AJ. Predictive factors for hepatocellular carcinoma in type 1 autoimmune hepatitis. Am J Gastroenterol. (2008) 103:1944–51. 10.1111/j.1572-0241.2008.01922.x 18564111

[46] Czaja AJ. Emerging therapeutic biomarkers of autoimmune hepatitis and their impact on current and future management. Expert Rev Gastroenterol Hepatol. (2018) 12:547–64. 10.1080/17474124.2018.1453356 29540068

[47] Ambros V. The functions of animal microRNAs. Nature. (2004) 431:350–5. 10.1038/nature02871 15372042

[48] Yamaura Y Tatsumi N Takagi S Tokumitsu S Fukami T Tajiri K Serum microRNA profiles in patients with chronic hepatitis B, chronic hepatitis C, primary biliary cirrhosis, autoimmune hepatitis, nonalcoholic steatohepatitis, or drug-induced liver injury. Clin Biochem. (2017) 50:1034–9. 10.1016/j.clinbiochem.2017.08.010 28823616

[49] Migita K Komori A Kozuru H Jiuchi Y Nakamura M Yasunami M. Circulating microRNA profiles in patients with type-1 autoimmune hepatitis. PLoS ONE. (2015) 10:e0136908. 10.1371/journal.pone.0136908 26575387 PMC4648542

[50] Halasz T Horvath G Par G Werling K Kiss A Schaff Z. miR-122 negatively correlates with liver fibrosis as detected by histology and FibroScan. World J Gastroenterol. (2015) 21:7814–23. 10.3748/wjg.v21.i25.7814 26167081 PMC4491968

[51] McKinney EF Lee JC Jayne DR Lyons PA Smith KG. T-cell exhaustion, co-stimulation and clinical outcome in autoimmunity and infection. Nature. (2015) 523:612–6. 10.1038/nature14468 26123020 PMC4623162

[52] Mataki N Kikuchi K Kawai T Higashiyama M Okada Y Kurihara C. Expression of PD-1, PD-L1, and PD-L2 in the liver in autoimmune liver diseases. Am J Gastroenterol. (2007) 102:302–12. 10.1111/j.1572-0241.2006.00948.x 17311651

[53] Matsumoto K Miyake Y Matsushita H Ohnishi A Ikeda F Shiraha H. Anti-programmed cell death-1 antibody as a new serological marker for type 1 autoimmune hepatitis. J Gastroenterol Hepatol. (2014) 29:110–5. 10.1111/jgh.12340 23869988

[54] Miyake Y Yamamoto K Matsushita H Abe M Takahashi A Umemura T. Multicenter validation study of anti-programmed cell death-1 antibody as a serological marker for type 1 autoimmune hepatitis. Hepatol Res. (2014) 44:1299–307. 10.1111/hepr.12305 24506172

[55] Aarslev K Dige A Greisen SR Kreutzfeldt M Jessen N Vilstrup H. Soluble programmed death-1 levels are associated with disease activity and treatment response in patients with autoimmune hepatitis. Scand J Gastroenterol. (2017) 52:93–9. 10.1080/00365521.2016.1233576 27604386

[56] Calandra T Bernhagen J Metz CN Spiegel LA Bacher M Donnelly T. MIF as a glucocorticoid-induced modulator of cytokine production. Nature. (1995) 377:68–71. 10.1038/377068a0 7659164

[57] Assis DN Leng L Du X Zhang CK Grieb G Merk M.. The role of macrophage migration inhibitory factor in autoimmune liver disease. Hepatology. (2014) 59:580–91. 10.1002/hep.26664 23913513 PMC3877200

[58] Assis DN Takahashi H Leng L Zeniya M Boyer JL Bucala R. A macrophage migration inhibitory factor polymorphism is associated with autoimmune hepatitis severity in US and Japanese patients. Dig Dis Sci. (2016) 61:3506–12. 10.1007/s10620-016-4322-z 27696094 PMC5106299

[59] Kristiansen M Graversen JH Jacobsen C Sonne O Hoffman HJ Law SK. Identification of the haemoglobin scavenger receptor. Nature. (2001) 409:198–201. 10.1038/35051594 11196644

[60] Gronbaek H Kreutzfeldt M Kazankov K Jessen N Sandahl T Hamilton-Dutoit S. Single-centre experience of the macrophage activation marker soluble (s)CD163 -associations with disease activity and treatment response in patients with autoimmune hepatitis. Aliment Pharmacol Ther. (2016) 44:1062–70. 10.1111/apt.13801 27679428

[61] Kazankov K Barrera F Moller HJ Bibby BM Vilstrup H George J. Soluble CD163, a macrophage activation marker, is independently associated with fibrosis in patients with chronic viral hepatitis B and C. Hepatology. (2014) 60:521–30. 10.1002/hep.27129 24623375

[62] Schneider P MacKay F Steiner V Hofmann K Bodmer JL Holler N. BAFF, a novel ligand of the tumor necrosis factor family, stimulates B cell growth. J Exp Med. (1999) 189:1747. 10.1084/jem.189.11.1747 10359578 PMC2193079

[63] Migita K Abiru S Maeda Y Nakamura M Komori A Ito M. Elevated serum BAFF levels in patients with autoimmune hepatitis. Hum Immunol. (2007) 68:586–91. 10.1016/j.humimm.2007.03.010 17584580

[64] Cookson S Constantini PK Clare M Underhill JA Bernal W Czaja AJ. Frequency and nature of cytokine gene polymorphisms in type 1 autoimmune hepatitis. Hepatology. (1999) 30:851–6. 10.1002/hep.510300412 10498633

[65] Czaja AJ Cookson S Constantini PK Clare M Underhill JA Donaldson PT. Cytokine polymorphisms associated with clinical features and treatment outcome in type 1 autoimmune hepatitis. Gastroenterology. (1999) 117:645–52. 10.1016/S0016-5085(99)70458-0 10464141

[66] Akberova D Kiassov AP Abdulganieva D. Serum cytokine levels and their relation to clinical features in patients with autoimmune liver diseases. J Immunol Res. (2017) 2017:9829436. 10.1155/2017/9829436 28299346 PMC5337382

[67] Qin B Li J Liang Y Yang Z Zhong R The association between Cytotoxic T lymphocyte associated antigen-4, Fas, tumor necrosis factor-α gene polymorphisms and autoimmune hepatitis: a meta-analysis. Dig Liver Dis Jun. (2014) 46:541–8. 10.1016/j.dld.2014.02.003 24629822

[68] Weiler-Normann C Schramm C Quaas A Wiegard C Glaubke C Pannicke N.. Infliximab as a rescue treatment in difficult-to-treat autoimmune hepatitis. J Hepatol. (2013) 58:529–34. 10.1016/j.jhep.2012.11.010 23178709

[69] Fujii K Rokutanda R Osugi Y Koyama Y Ota T. Adult-onset Still's disease complicated by autoimmune hepatitis: successful treatment with infliximab. Intern Med. (2012) 51:1125–8. 10.2169/internalmedicine.51.6824 22576401

[70] Rajanayagam J Lewindon PJ. Infliximab as rescue therapy in paediatric autoimmune hepatitis. J Hepatol. (2013) 59:908–9. 10.1016/j.jhep.2013.05.046 23792030

[71] Nedelkopoulou N Vadamalayan B Vergani D Mieli-Vergani G. Anti-TNFα treatment in children and adolescents with combined inflammatory bowel disease and autoimmune liver disease. J Pediatr Gastroenterol Nutr. (2018) 66:100–5. 10.1097/MPG.0000000000001759 28953529

[72] Atzeni F Talotta R Salaffi F Cassinotti A Varisco V Battellino M Immunogenicity and autoimmunity during anti-TNF-therapy. Autoimmun Rev. (2013) 13:703–8. 10.1016/j.autrev.2012.10.021 23207283

[73] Chen X Oppenheim JJ. Contrasting effects of TNF and anti-TNF on activation of effector T cells and regulatory T cells in autoimmunity. FEBS Lett. (2011) 585:3611–8. 10.1016/j.febslet.2011.04.025 21513711 PMC3164898

[74] Zheng L Fisher G Miller RE Peschon J Lynch DH Lenardo MJ. Induction of apoptosis in mature T cells by tumour necrosis factor. Nature. (1995) 377:348–51. 10.1038/377348a0 7566090

[75] Lopetuso LR Mocci G Marzo M D'Aversa F Rapaccini GL Guidi L. Harmful effects and potential benefits of Anti-Tumor Necrosis Factor (TNF)-α on the liver. Int J Mol Sci. (2018) 19:E2199. 10.3390/ijms19082199 30060508 PMC6121684

[76] Ricciuto A Kamath BM Walters TD Frost K Carman N Church PC. New onset autoimmune hepatitis during anti-tumor necrosis factor-alpha treatment in children. J Pediatr. (2018) 194:128–35.e1. 10.1016/j.jpeds.2017.10.071 29274889

[77] Faustman DL. TNF, TNF inducers, and TNFR2 agonists: a new path to type 1 diabetes treatment. Diabetes Metab Res Rev. (2018) 34. 10.1002/dmrr.2941 28843039

[78] Webb GJ Hirschfield GM Krawitt EL Gershwin ME Cellular and molecular mechanisms of autoimmune hepatitis, *Annu*. Rev. Pathol. (2018) 13:247–92. 10.1146/annurev-pathol-020117-043534 29140756

[79] De Biasio MB Periolo N Avagnina A García de Dávila MT Ciocca M Goñi J.. Liver infiltrating mononuclear cells in children with type 1 autoimmune hepatitis. J Clin Pathol. (2006) 59:417–23. 10.1136/jcp.2005.028613 16489183 PMC1860380

[80] Wen L Peakman M Lobo-Yeo A McFarlane BM Mowat AP Mieli-Vergani G. T-cell-directed hepatocyte damage in autoimmune chronic active hepatitis. Lancet. (1990) 336:1527–30. 10.1016/0140-6736(90)93306-A 1979365

[81] Bowen DG Warren A Davis T Hoffmann MW McCaughan GW Fazekas de St Groth B.. Cytokine-dependent bystander hepatitis due to intrahepatic murine CD8 T-cell activation by bone marrow-derived cells. Gastroenterology. (2002) 123:1252–64. 10.1053/gast.2002.36058 12360486

[82] Vergani D Mieli-Vergani G Mondelli M Portmann B Eddleston AL. Immunoglobulin on the surface of isolated hepatocytes is associated with antibody-dependent cell-mediated cytotoxicity and liver damage. Liver. (1987) 7:307–5. 10.1111/j.1600-0676.1987.tb00361.x 3437792

[83] Hardtke-Wolenski M Fischer K Noyan F Schlue J Falk CS Stahlhut M. Genetic predisposition and environmental danger signals initiate chronic autoimmune hepatitis driven by CD4+ T cells. Hepatology. (2013) 58:718–28. 10.1002/hep.26380 23475565

[84] Lohr HF Schlaak JF Lohse AW Bocher WO Arenz M Gerken G. Autoreactive CD4+ LKM-specific and anticlonotypic T-cell responses in LKM-1 antibody-positive autoimmune hepatitis. Hepatology. (1996) 24:1416–21. 10.1053/jhep.1996.v24.pm0008938173 8938173

[85] Ma Y Bogdanos DP Hussain MJ Underhill J Bansal S Longhi MS. Polyclonal T-cell responses to cytochrome P450IID6 are associated with disease activity in autoimmune hepatitis type 2. Gastroenterology. (2006) 130:868–82. 10.1053/j.gastro.2005.12.020 16530525

[86] Beland K Marceau G Labardy A Bourbonnais S Alvarez F. Depletion of B cells induces remission of autoimmune hepatitis in mice through reduced antigen presentation and help to T cells. Hepatology. (2015) 62:1511–23. 10.1002/hep.27991 26175263

[87] Liu X Jiang X Liu R Wang L Qian T Zheng Y. B cells expressing CD11b effectively inhibit CD4+ T-cell responses and ameliorate experimental autoimmune hepatitis in mice. Hepatology. (2015) 62:1563–75. 10.1002/hep.28001 26207521

[88] Boross P Leusen JH. Mechanisms of action of CD20 antibodies. Am J Cancer Res. (2012) 2:676–90. 23226614 PMC3512181

[89] Burak KW Swain MG Santodomingo-Garzon T Lee SS Urbanski SJ Aspinall AI Rituximab for the treatment of patients with autoimmune hepatitis who are refractory or intolerant to standard therapy. Can J Gastroenterol. (2013) 27:273–80. 10.1155/2013/512624 23712302 PMC3735730

[90] Al-Busafi SA Michel RP Deschenes M. Rituximab for refractory autoimmune hepatitis: a case report. Arab J Gastroenterol. (2013) 14:135–8. 10.1016/j.ajg.2013.08.009 24206745

[91] Rubin JN Te HS. Refractory autoimmune hepatitis: beyond standard therapy. Dig Dis Sci. (2016) 61:1757–62. 10.1007/s10620-015-4022-0 26725067

[92] Evans JT Shepard MM Oates JC Self SE Reuben A. Rituximab-responsive cryoglobulinemic glomerulonephritis in a patient with autoimmune hepatitis. J Clin Gastroenterol. (2008) 42:862–3. 10.1097/MCG.0b013e3180f60b7a 18458643

[93] Barth E Clawson J. A case of autoimmune hepatitis treated with rituximab. Case Rep Gastroenterol. (2010) 4:502–9. 10.1159/000322693 21151634 PMC2999734

[94] Santos ES Arosemena LR Raez LE O'Brien C Regev A. Successful treatment of autoimmune hepatitis and idiopathic thrombocytopenic purpura with the monoclonal antibody, rituximab: case report and review of literature. Liver Int. (2006) 26:625–9. 10.1111/j.1478-3231.2006.01262.x 16762009

[95] D'Agostino D Costaguta A Álvarez F. Successful treatment of refractory autoimmune hepatitis with rituximab. Pediatrics. (2013) 132:e526–30. 10.1542/peds.2011-1900 23821693

[96] Carey EJ Somaratne K Rakela J. Successful rituximab therapy in refractory autoimmune hepatitis and Evans syndrome. Rev Med Chil. (2011) 139:1484–7. 10.4067/S0034-98872011001100015 22446656

[97] Liberal R de Boer YS Andrade RJ Bouma G Dalekos GN Floreani A International Autoimmune Hepatitis Group (IAIHG). Expert clinical management of autoimmune hepatitis in the real world Aliment. Pharmacol Ther. (2017) 45:723–32. 10.1111/apt.13907 28004405

[98] Than NN Schmidt D Hodson J Wawman R Burak K Botter M Rituximab treatment experience in patients with complicated type 1 autoimmune hepatitis in Europe and North America. J Hepatol. (2018) 68:S217–8. 10.1016/S0168-8278(18)30652-4

[99] Carson KR Focosi D Major EO Petrini M Richey EA West DP. Monoclonal antibody-associated progressive multifocal leucoencephalopathy in patients treated with rituximab, natalizumab, and efalizumab: a Review from the Research on Adverse Drug Events and Reports (RADAR). Project Lancet Oncol. (2009) 10:816–24. 10.1016/S1470-2045(09)70161-5 19647202

[100] Ferraccioli G Gremese E. B cell activating factor (BAFF) and BAFF receptors: fakes and facts. Clin Exp Immunol. (2017) 190:291–2. 10.1111/cei.13039 28834574 PMC5680055

[101] Harrington LE Hatton RD Mangan PR Turner H Murphy TL Murphy KM. Interleukin 17-producing CD4 effector T cells develop via a lineage distinct from the T helper type 1 and 2 lineages. Nat Immunol. (2005) 6:1123–32. 10.1038/ni1254 16200070

[102] Korn T Bettelli E Oukka M Kuchroo VK. IL-17 and Th17 Cells. Annu Rev Immunol. (2009) 27:485–517. 10.1146/annurev.immunol.021908.132710 19132915

[103] Bettelli E Carrier Y Gao W Korn T Strom TB Oukka M. Reciprocal developmental pathways for the generation of pathogenic effector TH17 and regulatory T cells. Nature. (2006) 441:235–8. 10.1038/nature04753 16648838

[104] Wolk K Witte E Witte K Warszawska K Sabat R. Biology of interleukin-22. Semin Immunopathol. (2010) 32:17–31. 10.1007/s00281-009-0188-x 20127093

[105] Dardalhon V Korn T Kuchroo VK Anderson AC. Role of Th1 and Th17 cells in organ-specific autoimmunity. J Autoimmun. (2008) 31:252–6. 10.1016/j.jaut.2008.04.017 18502610 PMC3178062

[106] Zhao L Tang Y You Z Wang Q Liang S Han X. Interleukin-17 contributes to the pathogenesis of autoimmune hepatitis through inducing hepatic interleukin-6 expression. PLoS ONE. (2011) 6:e18909. 10.1371/journal.pone.0018909 21526159 PMC3079758

[107] Lobo-Yeo A Senaldi G Portmann B Mowat AP Mieli-Vergani G Vergani D. Class I and class II major histocompatibility complex antigen expression on hepatocytes: a study in children with liver disease. Hepatology. (1990) 12:224–32. 10.1002/hep.1840120208 2118117

[108] Zhang H Bernuzzi F Lleo A Ma X Invernizzi P. Therapeutic potential of IL-17-mediated signaling pathway in autoimmune liver diseases. Mediat Inflamm. (2015) 2015:436450. 10.1155/2015/436450 26146463 PMC4471389

[109] Longhi MS Liberal R Holder B Robson SC Ma Y Mieli-Vergani G. Inhibition of interleukin-17 promotes differentiation of CD25(-) cells into stable T regulatory cells in patients with autoimmune hepatitis. Gastroenterology. (2012) 142:1526–35. 10.1053/j.gastro.2012.02.041 22387392

[110] McInnes IB Mease PJ Ritchlin CT Rahman P Gottlieb AB Kirkham B. Secukinumab sustains improvement in signs and symptoms of psoriatic arthritis: 2 year results from the phase 3 FUTURE 2 study. Rheumatology. (2017) 56:1993–2003. 10.1093/rheumatology/kex301 28968735 PMC5850284

[111] Strober BE Germino R Guana A Greenberg JD Litman HJ Guo N US real-world effectiveness of secukinumab for the treatment of psoriasis: 6-month analysis from the Corrona Psoriasis Registry. J Dermatolog Treat. (2019) 29:1–9. 10.1080/09546634.2019.1603361 31035822

[112] Genovese MC Durez P Richards HB Supronik J Dokoupilova E Mazurov V. Efficacy and safety of secukinumab in patients with rheumatoid arthritis: a phase II, dose-finding, double-blind, randomised, placebo controlled study. Ann Rheum Dis. (2013) 72:863–9. 10.1136/annrheumdis-2012-201601 22730366

[113] Baeten D Baraliakos X Braun J Sieper J Emery P van der Heijde D. Anti-interleukin-17A monoclonal antibody secukinumab in treatment of ankylosing spondylitis: a randomised, double-blind, placebo-controlled trial. Lancet. (2013) 382:1705–13. 10.1016/S0140-6736(13)61134-4 24035250

[114] Dick AD Tugal-Tutkun I Foster S Zierhut M Melissa Liew SH Bezlyak V. Secukinumab in the treatment of noninfectious uveitis: results of three randomized, controlled clinical trials. Ophthalmology. (2013) 120:777–87. 10.1016/j.ophtha.2012.09.040 23290985

[115] Hueber W Sands BE Lewitzky S Vandemeulebroecke M Reinisch W Higgins PD Secukinumab in Crohn's Disease Study Group. Secukinumab, a human anti-IL-17A monoclonal antibody, for moderate to severe Crohn's disease: unexpected results of a randomised, double-blind placebo-controlled trial. Gut. (2012) 61:1693–700. 10.1136/gutjnl-2011-301668 22595313 PMC4902107

[116] Ritchlin C Rahman P Kavanaugh A McInnes IB Puig L Li S PSUMMIT 2 Study Group. Efficacy and safety of the anti-IL-12/23 p40 monoclonal antibody, ustekinumab, in patients with active psoriatic arthritis despite conventional non-biological and biological anti-tumour necrosis factor therapy: 6-month and 1-year results of the phase 3, multicentre, double-blind, placebo-controlled, randomised PSUMMIT 2 trial. Ann Rheum Dis. (2014) 73:990–9. 10.1136/annrheumdis-2013-204655 24482301 PMC4033144

[117] Sandborn WJ Gasink C Gao LL Blank MA Johanns J Guzzo C CERTIFI Study Group. Ustekinumab induction and maintenance therapy in refractory Crohn's disease. N Engl J Med. (2012) 367:1519–28. 10.1056/NEJMoa1203572 23075178

[118] Zweegers J Groenewoud JMM van den Reek JMPA Otero ME van de Kerkhof PCM Driessen RJB. Comparison of the 1- and 5-year effectiveness of adalimumab, etanercept and ustekinumab in patients with psoriasis in daily clinical practice: results from the prospective BioCAPTURE registry. Br J Dermatol. (2017) 176:1001–9. 10.1111/bjd.15023 27579864

[119] Mirouse A Barete S Desbois AC Comarmond C Sène D Domont F. Long term outcome of ustekinumab therapy for Behçet's disease. Arthritis Rheumatol. (2019). 10.1002/art.40912 [Epub ahead of print]. 31008548

[120] Segal BM Constantinescu CS Raychaudhuri A Kim L Fidelus-Gort R Kasper LH. Repeated subcutaneous injections of IL12/23 p40 neutralising antibody, ustekinumab, in patients with relapsing-remitting multiple sclerosis: a phase II, double-blind, placebo-controlled, randomised, dose-ranging study. Lancet Neurol. (2008) 7:796–804. 10.1016/S1474-4422(08)70173-X 18703004

[121] Hirschfield GM Gershwin ME Strauss R Mayo MJ Levy C Zou B Ustekinumab for patients with primary biliary cholangitis who have an inadequate response to ursodeoxycholic acid: a proof-of-concept study*, Hepatology*. (2016) 64:189–99. 10.1002/hep.28359 26597786

[122] Tonini A Gualtieri B Panduri S Romanelli M Chiricozzi A. A new class of biologic agents facing the therapeutic paradigm in psoriasis: anti-IL-23 agents. Expert Opin Biol Ther. (2018) 18:135–48. 10.1080/14712598.2018.1398729 29103330

[123] Shetty A Hanson R Korsten P Shawagfeh M Arami S Volkov S. Tocilizumab in the treatment of rheumatoid arthritis and beyond. Drug Des Devel Ther. (2014) 8:349–64. 10.2147/DDDT.S41437 24729685 PMC3974690

[124] Frampton JE. Tocilizumab: a review of its use in the treatment of juvenile idiopathic arthritis. Pediatric Drugs. (2013) 15:515–31. 10.1007/s40272-013-0053-1 24155139

[125] González-Gay MÁ Pina T Prieto-Peña D Calderon-Goercke M Gualillo O Castañeda S. Treatment of giant cell arteritis. Biochem Pharmacol. (2019) 165:230–9. 10.1016/j.bcp.2019.04.027 31034796

[126] Schwartz DM Kanno Y Villarino A Ward M Gadina M O'Shea JJ. JAK inhibition as a therapeutic strategy for immune and inflammatory diseases. Nat Rev Drug Discov. (2017) 16:843–62. 10.1038/nrd.2017.201 29104284

[127] Yang XO Pappu BP Nurieva R Akimzhanov A Kang HS Chung Y. T helper 17 lineage differentiation is programmed by orphan nuclear receptors ROR alpha and ROR gamma. Immunity. (2008) 28:29–39. 10.1016/j.immuni.2007.11.016 18164222 PMC2587175

[128] Khan PM El-Gendy Bel D Kumar N Garcia-Ordonez R Lin L Ruiz CH Small molecule amides as potent ROR-γ selective modulators Bioorg. Med Chem Lett. (2013) 23:532–6. 10.1016/j.bmcl.2012.11.025 PMC3534870 23232056

[129] Tang Q Bluestone JA. The Foxp3+ regulatory T cell: a jack of all trades, master of regulation. Nat Immunol. (2008) 9:239–44. 10.1038/ni1572 18285775 PMC3075612

[130] Josefowicz SZ Lu LF Rudensky AY. Regulatory T cells: mechanisms of differentiation and function. Annu Rev Immunol. (2012) 30:531–64. 10.1146/annurev.immunol.25.022106.141623 22224781 PMC6066374

[131] Hori S Nomura T Sakaguchi S. Control of regulatory T cell development by the transcription factor Foxp3. Science. (2003) 299:1057–61. 10.1126/science.1079490 12522256

[132] Karim M Kingsley CI Bushell AR Sawitzki BS Wood KJ. Alloantigen-induced CD25+ CD4+ regulatory T cells can develop *in vivo* from CD25-CD4? precursors in a thymusin-dependent process. J Immunol. (2004) 172:923–8. 10.4049/jimmunol.172.2.923 14707064

[133] Fletcher JM Lonergan R Costelloe L Kinsella K Moran B O'Farrelly C. CD39+Foxp3+ regulatory T Cells suppress pathogenic Th17 cells and are impaired in multiple sclerosis. J Immunol. (2009) 183:7602–10. 10.4049/jimmunol.0901881 19917691

[134] O'Garra A Vieira PL Vieira P Goldfeld AE. IL-10-producing and naturally occurring CD4+ Tregs: limiting collateral damage. J Clin Invest. (2004) 114:1372–8. 10.1172/JCI200423215 15545984 PMC525746

[135] Kobie JJ Shah PR Yang L Rebhahn JA Fowell DJ Mosmann TR. T regulatory and primed uncommitted CD4 T cells express CD73, which suppresses effector CD4 T cells by converting 5'-adenosine monophosphate to adenosine. J Immunol. (2006) 177:6780–6. 10.4049/jimmunol.177.10.6780 17082591

[136] Liberal R Grant CR Holder BS Ma Y Mieli-Vergani G Vergani D. The impaired immune regulation of autoimmune hepatitis is linked to a defective galectin-9/tim-3 pathway. Hepatology. (2012) 56:677–86. 10.1002/hep.25682 22371007

[137] Okeke EB Uzonna JE. The pivotal role of regulatory T cells in the regulation of innate immune cells. Front Immunol. (2019) 10:680. 10.3389/fimmu.2019.00680 31024539 PMC6465517

[138] Zhao B Chang L Fu H Sun G Yang W. The Role of Autoimmune Regulator (AIRE) in Peripheral Tolerance. J Immunol Res. (2018) 2018:3930750. 10.1155/2018/3930750 30255105 PMC6142728

[139] Van der Vliet HJ Nieuwenhuis EE IPEX as a result of mutations in FOXP3. Clin Dev Immunol. (2007) 2007:89017 10.1155/2007/89017 18317533 PMC2248278

[140] Wan YY Flavell RA. Regulatory T-cell functions are subverted and converted owing to attenuated Foxp3 expression. Nature. (2007) 445:766–70. 10.1038/nature05479 17220876

[141] Piloni D Morosini M Magni S Balderacchi A Scudeller L Cova E. Analysis of long term CD4+CD25highCD127- T-reg cells kinetics in peripheral blood of lung transplant recipients. BMC Pulm Med. (2017) 17:102. 10.1186/s12890-017-0446-y 28720146 PMC5516333

[142] Longhi MS Ma Y Bogdanos DP Cheeseman P Mieli-Vergani G Vergani D. Impairment of CD4(+)CD25(+) regulatory T-cells in autoimmune liver disease. J Hepatol. (2004) 41:31–7. 10.1016/j.jhep.2004.03.008 15246204

[143] Longhi MS Hussain MJ Mitry RR Arora SK Mieli-Vergani G Vergani D. Functional study of CD4+CD25+ regulatory T cells in health and autoimmune hepatitis. J Immunol. (2006) 176:4484–91. 10.4049/jimmunol.176.7.4484 16547287

[144] Ferri S Longhi MS De Molo C Lalanne C Muratori P Granito A. A multifaceted imbalance of T cells with regulatory function characterizes type 1 autoimmune hepatitis. Hepatology. (2010) 52:999–1007. 10.1002/hep.23792 20683931

[145] Grant CR Liberal R Holder BS Cardone J Ma Y Robson SC. Dysfunctional CD39(POS) regulatory T cells and aberrant control of T-helper type 17 cells in autoimmune hepatitis. Hepatology. (2014) 59:1007–15. 10.1002/hep.26583 23787765 PMC6377365

[146] Liberal R Grant CR Holder BS Cardone J Martinez-Llordella M Ma Y. In autoimmune hepatitis type 1 or the autoimmune hepatitis-sclerosing cholangitis variant defective regulatory T-cell responsiveness to IL-2 results in low IL-10 production and impaired suppression. Hepatology. (2015) 62:863–75. 10.1002/hep.27884 25953611

[147] Peiseler M Sebode M Franke B Wortmann F Schwinge D Quaas A FOXP3+ regulatory T cells in autoimmune hepatitis are fully functional and not reduced in frequency. J Hepatol. (2012) 57:125–32. 10.1016/j.jhep.2012.02.029 22425700

[148] Diestelhorst J Junge N Schlue J Falk CS Manns MP Baumann U. Pediatric autoimmune hepatitis shows a disproportionate decline of regulatory T cells in the liver and of IL-2 in the blood of patients undergoing therapy. PLoS ONE. (2017) 12:e0181107. 10.1371/journal.pone.0181107 28700730 PMC5507441

[149] Jeffery HC Braitch MK Brown S Oo YH. Clinical potential of regulatory T cell therapy in liver diseases: an overview and current perspectives. Front Immunol. (2016) 7:334. 10.3389/fimmu.2016.00334 27656181 PMC5012133

[150] Lapierre P Beland K Yang R Alvarez F. Adoptive transfer of *ex vivo* expanded regulatory T cells in an autoimmune hepatitis murine model restores peripheral tolerance. Hepatology. (2013) 57:217–27. 10.1002/hep.26023 22911361

[151] Oo YH Weston CJ Lalor PF Curbishley SM Withers DR Reynolds GM. Distinct roles for CCR4 and CXCR3 in the recruitment and positioning of regulatory T cells in the inflamed human liver. J Immunol. (2010) 184:2886–98. 10.4049/jimmunol.0901216 20164417

[152] Todo S Yamashita K Goto R Zaitsu M Nagatsu A Oura T. A pilot study of operational tolerance with a regulatory T cell-based cell therapy in living donor liver transplantation. Hepatology. (2016) 64:632–43. 10.1002/hep.28459 26773713

[153] Trzonkowski P Bieniaszewska M Juścinska J Dobyszuk A Krzystyniak A Marek N. First-in-man clinical results of the treatment of patients with graft versus host disease with human *ex vivo* expanded CD4+CD25+CD127 – T regulatory cells. Clin Immunol. (2009) 133:22–6. 10.1016/j.clim.2009.06.001 19559653

[154] Di Ianni M Falzetti F Carotti A Terenzi A Castellino F Bonifacio E. Tregs prevent GVHD and promote immune reconstitution in HLA-haploidentical transplantation. Blood. (2011) 117:3921–8. 10.1182/blood-2010-10-311894 21292771

[155] Brunstein CG Miller JS Cao Q McKenna DH Hippen KL Curtsinger J. Infusion of *ex vivo* expanded T regulatory cells in adults transplanted with umbilical cord blood: safety profile and detection kinetics. Blood. (2011) 117:1061–70. 10.1182/blood-2010-07-293795 20952687 PMC3035067

[156] Marek-Trzonkowska N Mysliwiec M Dobyszuk A Grabowska M Techmanska I Juscinska J. Administration of CD4+CD25highCD127 – regulatory T cells preserves beta-cell function in type 1 diabetes in children. Diabetes Care. (2012) 35:1817–20. 10.2337/dc12-0038 22723342 PMC3425004

[157] Bluestone JA Buckner JH Fitch M Gitelman SE Gupta S Hellerstein MK. Type 1 diabetes immunotherapy using polyclonal regulatory T cells. Sci Transl Med. (2015) 7:315ra189. 10.1126/scitranslmed.aad4134 26606968 PMC4729454

[158] Tang Q Henriksen KJ Bi M Finger EB Szot G Ye J. *In vitro*-expanded antigen-specific regulatory T cells suppress autoimmune diabetes. J Exp Med. (2004) 199:1455–65. 10.1084/jem.20040139 15184499 PMC2211775

[159] Tarbell KV Yamazaki S Olson K Toy P Steinman RM. CD25+ CD4+ T cells, expanded with dendritic cells presenting a single autoantigenic peptide, suppress autoimmune diabetes. J Exp Med. (2004) 199:1467–77. 10.1084/jem.20040180 15184500 PMC2211787

[160] Brusko TM Koya RC Zhu S Lee MR Putnam AL McClymont SA. Human antigen-specific regulatory T cells generated by T cell receptor gene transfer. PLoS ONE. (2010) 5:e11726. 10.1371/journal.pone.0011726 20668510 PMC2908680

[161] Noyan F Lee YS Zimmermann K Hardtke-Wolenski M Taubert R Warnecke G. Isolation of human antigen-specific regulatory T cells with high suppressive function. Eur J Immunol. (2014) 44:2592–602. 10.1002/eji.201344381 24990119

[162] Noyan F Lee YS Hardtke-Wolenski M Knoefel AK Taubert R Baron U. Donor-specific regulatory T cells generated on donor B cells are superior to CD4+CD25high cells in controlling alloimmune responses in human-ized mice. Transplant Proc. (2013) 45:1832–7. 10.1016/j.transproceed.2013.01.073 23769053

[163] Cheraï M Hamel Y Baillou C Touil S Guillot-Delost M Charlotte F. Generation of human alloantigen-specific regulatory T cells under good manufacturing practice-compliant conditions for cell therapy. Cell Transplant. (2015) 24:2527–40. 10.3727/096368914X683566 25198125

[164] June CH O'Connor RS Kawalekar OU Ghassemi S Milone MC. CAR T cell immunotherapy for human cancer. Science. (2018) 359:1361–65. 10.1126/science.aar6711 29567707

[165] MacDonald KG Hoeppli RE Huang Q Gillies J Luciani DS Orban PC. Alloantigen-specific regulatory T cells generated with a chimeric antigen receptor. J Clin Invest. (2016) 126:1413–24. 10.1172/JCI82771 26999600 PMC4811124

[166] Boardman DA Philippeos C Fruhwirth GO Ibrahim MA Hannen RF Cooper D. Expression of a chimeric antigen receptor specific for donor HLA class I enhances the potency of human regulatory T cells in preventing human skin transplant rejection. Am J Transplant. (2017) 17:931–43. 10.1111/ajt.14185 28027623

[167] Longhi MS Hussain MJ Kwok WW Mieli-Vergani G Ma Y Vergani D. Autoantigen-specific regulatory T cells, a potential tool for immune-tolerance reconstitution in type-2 autoimmune hepatitis. Hepatology. (2011) 53:536–47. 10.1002/hep.24039 21274874

[168] Liao W Lin JX Leonard WJ. Interleukin-2 at the crossroads of effector responses, tolerance, and immunotherapy. Immunity. (2013) 38:13–25. 10.1016/j.immuni.2013.01.004 23352221 PMC3610532

[169] Wakabayashi K Lian ZX Moritoki Y Lan RY Tsuneyama K Chuang YH. IL-2 receptor alpha(-/-) mice and the development of primary biliary cirrhosis. Hepatology. (2006) 44:1240e−49. 10.1002/hep.21385 17058261

[170] Webb GJ Hirschfield GM. Using GWAS to identify genetic predisposition in hepatic autoimmunity. J Autoimmun. (2016) 66:25–39. 10.1016/j.jaut.2015.08.016 26347073

[171] Bo X Broome U Remberger M Sumitran-Holgersson S. Tumour necrosis factor alpha impairs function of liver derived T lymphocytes and natural killer cells in patients with primary sclerosing cholangitis. Gut. (2001) 49:131–41. 10.1136/gut.49.1.131 11413121 PMC1728361

[172] Czaja AJ Sievers C Zein NN. Nature and behavior of serum cytokines in type 1 autoimmune hepatitis. Dig Dis Sci. (2000) 45:1028–35. 10.1023/A:1005506031717 10795772

[173] Diestelhorst J Junge N Jonigk D Schlue J Falk CS Manns MP. Baseline IL-2 and the AIH score can predict the response to standard therapy in paediatric autoimmune hepatitis. Sci Rep. (2018) 8:419. 10.1038/s41598-017-18818-5 29323192 PMC5764983

[174] Hartemann A Bensimon G Payan CA Jacqueminet S Bourron O Nicolas N. Low-dose interleukin 2 in patients with type 1 diabetes: a phase 1/2 randomised, double-blind, placebo-controlled trial. Lancet Diabetes Endocrinol. (2013) 1:295–305. 10.1016/S2213-8587(13)70113-X 24622415

[175] Von Spee-Mayer C Siegert EE Abdirama D Rose A Klaus A Alexander T Low-dose interleukin-2 selectively corrects regulatory T cell defects in patients with systemic lupus erythematosus, *Ann*. Rheum Dis. (2016) 75:1407–15. 10.1136/annrheumdis-2015-207776 26324847

[176] Lim TY Martinez-Llordella M Kodela E Gray E Heneghan MA Sanchez-Fueyo A. Low dose interleukin-2 for refractory autoimmune hepatitis. Hepatology. (2018) 68:1649–52. 10.1002/hep.30059 29698571

[177] Long SA Rieck M Sanda S Bollyky JB Samuels PL Goland R. Rapamycin/IL-2 combination therapy in patients with type 1 diabetes augments Tregs yet transiently impairs beta-cell function. Diabetes. (2012) 61:2340–8. 10.2337/db12-0049 22721971 PMC3425404

[178] Trotta E Bessette PH Silveria SL Ely LK Jude KM Le DT. A human anti-IL-2 antibody that potentiates regulatory T cells by a structure-based mechanism. Nat Med. (2018) 24:1005–14. 10.1038/s41591-018-0070-2 29942088 PMC6398608

[179] Uccelli A Moretta L Pistoia V. Mesenchymal stem cells in health and disease. Nat Rev Immunol. (2008) 8:726–36. 10.1038/nri2395 19172693

[180] Arsenijevic A Harrell CR Fellabaum C Volarevic V. Mesenchymal stem cells as new therapeutic agents for the treatment of primary biliary cholangitis. Anal Cell Pathol. (2017) 2017:7492836. 10.1155/2017/7492836 29410945 PMC5749170

[181] Wang L Li J Liu H Li Y Fu J Sun Y. Pilot study of umbilical cord derived mesenchymal stem cell transfusion in patients with primary biliary cirrhosis. J Gastroenterol Hepatol. (2013) 28:85–92. 10.1111/jgh.12029 23855301

[182] Cantarelli C Angeletti A Cravedi P. Erythropoietin, a multifaceted protein with innate and adaptive immune modulatory activity. Am J Transplant. (2019) 19: 2407–14. 10.1111/ajt.15369 30903735 PMC6711804

[183] Lozupone CA Stombaugh JI Gordon JI Jansson JK Knight R. Diversity, stability and resilience of the human gut microbiota. Nature. (2012) 489:220–30. 10.1038/nature11550 22972295 PMC3577372

[184] Henao-Mejia J Elinav E Thaiss CA Licona-Limon P Flavell RA. Role of the intestinal microbiome in liver disease. J Autoimmun. (2013) 46:66–73. 10.1016/j.jaut.2013.07.001 24075647

[185] Strowig T Henao-Mejia J Elinav E Flavell R. Inflammasomes in health and disease. Nature. (2012) 481:278–86. 10.1038/nature10759 22258606

[186] Wen L Ley RE Volchkov PY Stranges PB Avanesyan L Stonebraker AC. Innate immunity and intestinal microbiota in the development of Type 1 diabetes. Nature. (2008) 455:1109–13. 10.1038/nature07336 18806780 PMC2574766

[187] Frank DN Robertson CE Hamm CM Kpadeh Z Zhang T Chen H. Disease phenotype and genotype are associated with shifts in intestinal-associated microbiota in inflammatory bowel diseases. Inflamm Bowel Dis. (2011) 17:179–84. 10.1002/ibd.21339 20839241 PMC3834564

[188] Vaahtovuo J Munukka E Korkeamaki M Luukkainen R Toivanen P. Fecal microbiota in early rheumatoid arthritis. J Rheumatol. (2008) 35:1500–5. 18528968

[189] Tabibian JH O'Hara SP Trussoni CE Tietz PS Splinter PL Mounajjed T. Absence of the intestinal microbiota exacerbates hepatobiliary disease in a murine model of primary sclerosing cholangitis. Hepatology. (2016) 63:185–96. 10.1002/hep.27927 26044703 PMC4670294

[190] Abe K Takahashi A Fujita M Imaizumi H Hayashi M Okai K. Dysbiosis of oral microbiota and its association with salivary immunological biomarkers in autoimmune liver disease. PLoS ONE. (2018) 13:e0198757. 10.1371/journal.pone.0198757 29969462 PMC6029758

[191] Yuksel M Wang Y Tai N Peng J Guo J Beland K. A novel humanized mouse model for autoimmune hepatitis and the association of gut microbiota with liver inflammation. Hepatology. (2015) 62:1536–50. 10.1002/hep.27998 26185095 PMC4763614

[192] Eksteen B Grant AJ Miles A Curbishley SM Lalor PF Hübscher SG. Hepatic endothelial CCL25 mediates the recruitment of CCR9+ gut-homing lymphocytes to the liver in primary sclerosing cholangitis. J Exp Med. (2004) 200:1511–7. 10.1084/jem.20041035 15557349 PMC2211943

[193] Markle JG Frank DN Adeli K von Bergen M Danska JS. Microbiome manipulation modifies sex-specific risk for autoimmunity. Gut Microbes. (2014) 5:485–93. 10.4161/gmic.29795 25007153

[194] Hsu MC Liu SH Wang CW Hu NY Wu ESC Shih YC. JKB-122 is effective, alone or in combination with prednisolone in Con A-induced hepatitis. Eur J Pharmacol. (2017) 812:113–20. 10.1016/j.ejphar.2017.07.012 28694068

[195] Relman DA. Microbiota: a high-pressure situation for bacteria. Nature. (2017) 551:571–2. 10.1038/nature24760 29143820

[196] Kleinewietfeld M Manzel A Titze J Kvakan H Yosef N Linker RA Sodium chloride drives autoimmune disease by the induction of pathogenic TH17cells. Nature. (2013) 496:518–22. 10.1038/nature11868 23467095 PMC3746493

[197] Smith PM Howitt MR Panikov N Michaud M Gallini CA Bohlooly YM The microbial metabolites, short-chain fatty acids, regulate colonic Tregs cell homeostasis. Science. (2013) 341:569–73. 10.1126/science.1241165 23828891 PMC3807819

[198] Hu ED Chen DZ Wu JL Lu FB Chen L Zheng MH. High fiber dietary and sodium butyrate attenuate experimental autoimmune hepatitis through regulation of immune regulatory cells and intestinal barrier. Cell Immunol. (2018) 328:24–32. 10.1016/j.cellimm.2018.03.003 29627063

[199] Lin R Zhou L Zhang J Wang B. Abnormal intestinal permeability and microbiota in patients with autoimmune hepatitis. Int J Clin Exp Pathol. (2015) 8:5153–60. 26191211 PMC4503083

